# Modifications of quinolones and fluoroquinolones: hybrid compounds and dual-action molecules

**DOI:** 10.1007/s00706-018-2215-x

**Published:** 2018-06-07

**Authors:** Joanna Fedorowicz, Jarosław Sączewski

**Affiliations:** Department of Organic Chemistry, Medical University of Gdańsk, Al. Gen. J. Hallera 107, 80-416 Gdańsk, Poland

**Keywords:** Antibiotics, Antitumor agents, Antiviral activity, Conjugates, Drug research, Hybrid drugs

## Abstract

**Abstract:**

This review is aimed to provide extensive survey of quinolones and fluoroquinolones for a variety of applications ranging from metal complexes and nanoparticle development to hybrid conjugates with therapeutic uses. The review covers the literature from the past 10 years with emphasis placed on new applications and mechanisms of pharmacological action of quinolone derivatives. The following are considered: metal complexes, nanoparticles and nanodrugs, polymers, proteins and peptides, NO donors and analogs, anionic compounds, siderophores, phosphonates, and prodrugs with enhanced lipophilicity, phototherapeutics, fluorescent compounds, triazoles, hybrid drugs, bis-quinolones, and other modifications. This review provides a comprehensive resource, summarizing a broad range of important quinolone applications with great utility as a resource concerning both chemical modifications and also novel hybrid bifunctional therapeutic agents.

**Graphical abstract:**

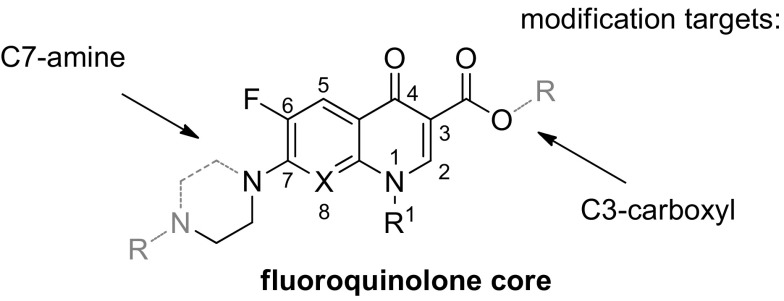

## Introduction

**Fig. 1:**
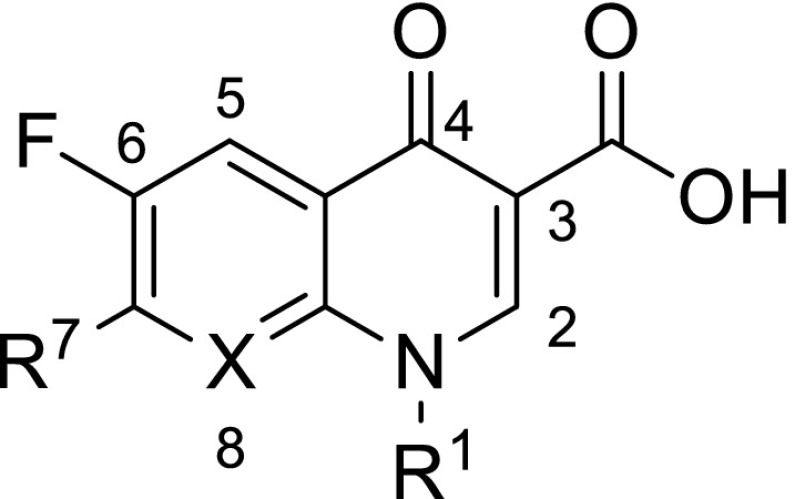
General structure of fluoroquinolones with atom numeration

Fluoroquinolones (Fig. 1) are broad-spectrum synthetic antibiotics (effective for both Gram-negative and Gram-positive bacteria) that play an important role in treatment of serious bacterial infections, especially hospital-acquired infections and others in which resistance to older antibacterial classes is suspected. Since the discovery of nalidixic acid by George Lesher in 1962 [1], over ten thousand analogs have been synthesized from which four generations of chemotherapeutics with broad spectrum of antibacterial activities have emerged [2]. Fluoroquinolones can enter cells easily via porins and, therefore, are often used to treat intracellular pathogens. Quinolone anti-microbial agents exert their antibacterial action via inhibition of homologous type II topoisomerases, DNA gyrase, and DNA topoisomerase IV [3]. The molecular basis for the quinolone inhibition mechanism has been extensively studied. A crystal structure of moxifloxacin in complex with *Acinetobacter baumannii* topoisomerase IV shows the wedge-shaped quinolone stacking between base pairs at the DNA cleavage site and binding conserved residues in the DNA cleavage domain through chelation of a noncatalytic magnesium ion [4]. The position 7 is considered to be one that directly interacts with DNA gyrase [5–8], or topoisomerase IV. The R^7^ substituent greatly influences potency, spectrum, and pharmacokinetics. A recent interesting observation is that increased bulkiness of R^7^ appears to confer protection from the efflux exporter proteins of bacteria, and diminishes the likelihood of bacterial resistance in wild-type bacterial strains [9–11], and increases anti-anaerobic activity.

In recent years, the concept of “dual-action drugs” has been gaining popularity in medicinal chemistry and medicine. Since a single drug is not always able to adequately control the illness, the combination of drugs with different pharmacotherapeutic profile may be needed [12]. Drugs involving the incorporation of two biologically active compounds in a single molecule with the intention of exerting dual drug action have been described [13]. For example, one of the hybrid parts may be incorporated to counterbalance the known side effects associated with the other hybrid part, or to amplify its effects through action on another biological target. In addition, hybrid drugs could be used to avoid fast developing bacterial resistance caused by frequent mutations in bacterial genome.

Interestingly, the fluoroquinolone chemotherapies linked to another antibacterial agent represent the most comprehensively described hybrid compounds. This review deals with the recent literature (2007–2017) concerning custom applications of quinolones and fluoroquinolones, as well as their hybrid conjugates with dual or enhanced action mechanisms.

## Metal complexes

Copper is one of the most important biometals due to its biological role and potential synergetic activity with drugs [14]. Cu(II) complexes with drugs are much more active in the presence of nitrogen-donor heterocyclic ligands, such as 2,2′-bipyridine, 1,10-phenanthroline, or 2,2′-dipyridylamine [15]. Hernández-Gil and coworkers reported the synthesis of two new ternary complexes of Cu(II) with ciprofloxacin and 1,10-phenanthroline. The aim of the study was to obtain artificial nucleases capable of cleaving DNA chains. The nucleolytic activity of copper complexes with nitrogen-donor heterocyclic ligand was revealed in the presence of H_2_O_2_ and reducing agents [16]. The chemical nuclease activity tests were performed in the presence of ascorbate and have shown that both complexes are efficient in DNA breaking. Mechanistic studies with various radical oxygen scavengers were undertaken and revealed that the cleavage reaction is mediated by hydroxyl radicals, superoxide anion, and singlet oxygen [17].

**Fig. 2:**
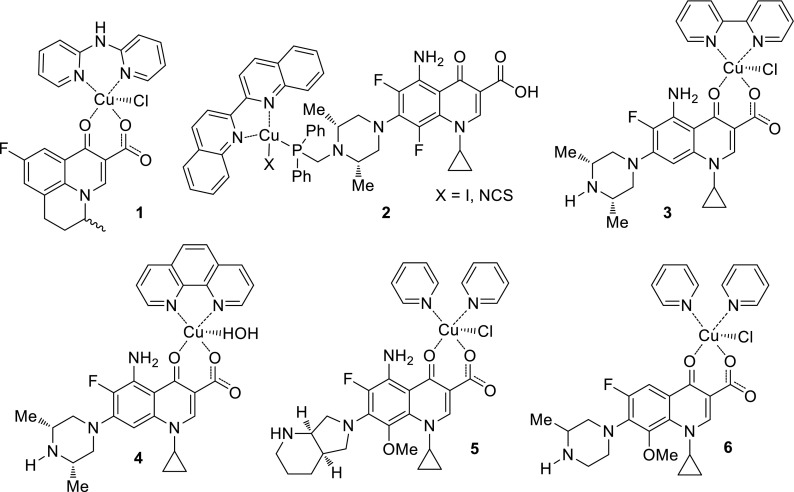
Structures of flumequine–Cu(II)-2,2′-bipyridylamine (**1**), sparfloxacin–Cu(I)-2,2′-biquinoline (**2**), sparfloxacin–Cu(II)-2,2′-bipyridine (**3**), sparfloxacin–Cu(II)-1,10-phenanthroline (**4**), moxifloxacin–Cu(II)-bipyridyl (**5**), and gatifloxacin–Cu(II)–bipyridyl (**6**) complexes

Chalkidou and coworkers designed a series of Cu(II) complexes with another quinolone antibiotic—flumequine. This synthetic drug belongs to the first generation of quinolones and is chiral. The complexes were prepared in the absence or the presence of the nitrogen-donor heterocyclic ligands: 2,2′-bipyridylamine (**1**), 2,2′-bipyridine, pyridine, or 1,10-phenanthroline (Fig. 2). In the resultant complexes, flumequine behaved as a deprotonated bidentate ligand being coordinated to copper via the pyridine oxygen and one carboxylate oxygen. All novel complexes showed higher affinity to bovine and human serum albumin (proteins involved in the transport of metal ions and metal–drug complexes through the blood stream) than free flumequine. Furthermore, the complexes exhibited similar or higher binding constants to calf-thymus DNA than free quinolone with the highest value for the complex with pyridine ligand. The mechanism of DNA binding probably involves intercalation, as inferred on the basis of hypochromic effect observed with UV spectroscopy [18].

Complexes of copper(I) iodide or copper(I) thiocyanate and phosphine derivative of sparfloxacin bearing auxiliary steric hindered diimine ligands (2,9-dimethyl-1,10-phenanthroline or 2,2′-biquinoline (**2**)) were prepared by Komarnicka and coworkers. Phosphine ligand was used to avoid oxidation and hydrolysis reactions by a strong copper–phosphine interaction. The conjugates obtained were tested against CT26 (mouse colon carcinoma) and A549 (human lung adenocarcinoma) cancer cell lines. The cytotoxicity of all compounds was found to be significantly increased (IC_50_ 6.04 ± 0.3–42.64 ± 0.73) in comparison with free sparfloxacin (IC_50_ 122.84 ± 4.21–273.50 ± 10.63) and extremely higher than cisplatin (IC_50_ 222.45 ± 10.78–298.12 ± 13.09) [19].

Neutral sparfloxacin–copper complexes were also utilized by Efthimiadou and coworkers. They prepared conjugates bearing ligands such as 2,2′-bipyridine (**3**), 1,10-phenanthroline, or 2,2′-dipyridylamine in high yields (65–70%) by the template reaction of equimolar quantities of the deprotonated sparfloxacin, CuCl_2_, and the corresponding N-donor ligand. The copper atom in obtained conjugates was five-coordinative and had slightly distorted square pyramidal geometry. Sparfloxacin was bound to Cu(II) via the pyridone and one carboxylate oxygens. The interactions of complexes with calf-thymus DNA showed that the complexes are able to bind DNA by intercalation mode. Antibacterial activity was tested against *Escherichia coli*, *Pseudomonas aeruginosa*, and *Staphylococcus aureus*. The conjugates were found to be more active than the parent drug against *E. coli*, but less active against remaining strains of bacteria with the lowest MIC values obtained for complexes bearing 2,2′-bipyridine and 1,10-phenanthroline ligands. These two complexes were tested as potential anticancer agents against human leukemia cell line HL-60 (peripheral blood human promyelocytic leukemia) in MTT assays and showed enhanced cytotoxic properties compared to free sparfloxacin which displayed no cytotoxic effect [20].

Shingnapurkar and coworkers also prepared sparfloxacin–Cu complexes having butterfly motif to expand fluoroquinolone activity on anti-proliferative properties against cancer cells. Fluoroquinolones are able to inhibit DNA topoisomerase in mammalian cells. This enzyme is overexpressed in hormone independent breast cancer cell lines. The complexes of fluoroquinolone and copper alone or with appended ancillary ligands, namely, 2,2′bipyridine, 1,10-phenanthroline (**4**), and 4,5-diazafluoren-9-one, were synthesized and characterized. The obtained conjugates were tested against BT20 breast cancer cell line IIα. IC_50_ values of novel complexes were four- to tenfold lower than in case of the parent drug indicating that anti-proliferative activity of quinolones may be related to their metal chelating ability. The dimeric compound of sparfloxacin and copper without additional ligands was the most potent molecule in the series [21].

Another research group synthesized moxifloxacin–copper complexes showing antitumor activity against breast cancer cells. They prepared four new conjugates, with or without additional ligands (pyridyl, bipyridyl (**5**), and phenanthroline), and performed anti-proliferative tests against estrogen-independent MDA-MB-231 and BT-20, as well as hormone-dependent MCF-7 and T47D cancer cell lines. All the conjugates were able to induce activity of caspases-3/7 and apoptosis in breast cancer cells with no toxic effect on MCF-10A, normal breast epithelial cell line. Moxifloxacin alone did not exhibit any anti-proliferative or apoptosis-inducing properties against any of the cell lines examined; however, when complexed with copper, it exhibited divergent cancer cell-specific activity with the strongest effect for phenanthroline adduct [22].

Complexes of copper and moxifloxacin or gatifloxacin bearing bipyridyl or phenanthroline ligands were also prepared by Singh and coworkers and tested in human lung carcinoma cells A-549. The highest cytotoxic activity exhibited complex **6** [gatifloxacin–Cu(II)-bipyridyl]. DNA fragmentation, cell shrinkage, transformation of cells into small membrane-bound vesicles or apoptotic bodies were observed in treated cells. Late apoptosis was perhaps induced by chromatin condensation. The metal complexes enhanced the apoptotic effect of the parent quinolone drugs, which may be useful for designing more effective drugs against lung cancer [23].

Technetium-99m is a radionuclide which serves as imaging agent because of its high biological stability [24], while rhenium is its non-radioactive analog possessing cytotoxic properties in some complexes [25]. Kydonaki and coworkers synthesized tricarbonyl complexes of Re(I) and ^99m^Tc with oxolinic acid or enrofloxacin in the presence of methanol (**7a**), triphenylphosphine (**7b**), or imidazole (**7c**) as coligands (Scheme 1). The resultant conjugates were neutral, air-stable, and DMSO-soluble, but insoluble in water and most organic solvents. The deprotonated quinolone ligands were bound bidentately to Re(I) ion through the pyridone oxygen and one carboxylate oxygen. Interaction with calf-thymus DNA was investigated by UV spectroscopy and affinity to bovine and human serum albumin was evaluated by fluorescence emission spectroscopy. Mode of interaction with nucleic acids was identified as intercalation and the highest DNA-binding constant was achieved for Re-enrofloxacin–methanol complex **7a**. The affinity to bovine and human serum albumin was similar or higher than that of free quinolones. Topoisomerase IIα inhibition experiments revealed that Re–enrofloxacin–imidazole complex displays ability to inhibit the enzyme at the concentration of 100 µM. This result suggests that metal coordination has a considerable impact on the activity of quinolones. The radiotracer complex of technetium, enrofloxacin, and imidazole was investigated in cellular uptake and biodistribution studies. The complex was able to enter K-562 human erythroleukemia cells and had been distributed in cellular compartments such as nuclei, mitochondria, and cytosol, with the highest accumulation in mitochondria. Notably, fast clearance from blood and muscle was observed after injection of the tracer conjugate in healthy mice, which indicate suitable pharmacokinetic profile for further evaluation as imaging agent [26].

**Figure:**
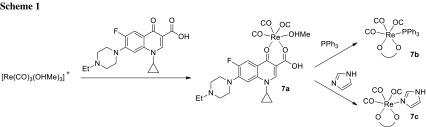

## Nanoparticles and nanodrugs

Biopolymer encapsulation of drug to form micro- and nanoparticles can be used as a drug delivery tool to change bioavailability, modify pharmacokinetics, target the drug, and redirect the antibiotic to tissues or organs, where infection occurs. Fluoroquinolones exhibit high affinity for binding Mg^2+^, which causes a depletion of the ion in bones and articular cartilage. The concentration of ofloxacin (fluoroquinolone widely used in hospitals) in the articular cartilage is three times higher than the corresponding concentration in plasma [27]. Lee and coworkers formed microparticles of albumin and hypromellose acetate succinate (HPMCAS) containing ofloxacin achieved by the spray dry method. Albumin was chosen, because it is biocompatible, biodegradable, and non-toxic natural protein component of blood [28]. HPMCAS is a hydrophilic cellulose derivative bearing succinyl groups and acts as entering coating agent. The obtained particles’ morphology was spherical with a smooth surface. Particle size (0.1–7 µm) depended on ofloxacin concentration. Ofloxacin nanospheres were administrated to BALB/c mice and good distribution was maintained. The release of ofloxacin was more sustained than ofloxacin in solution in all organs tested (spleen, brain, liver, and lung). This particle formulation is more favorable for treatment of diseases that affect the liver and brain, because the release from the particles was extended there by 24 and 48 h, respectively, and dosing regimens would be improved by less frequent dosing [29].

A different approach was used by Marslin and coworkers [30]. They used nanoparticles made of two different polymers, namely, poly(d,l-lactic-*co*-glycolic acid (PLGA) and methoxy poly(ethylene glycol)-b-poly(lactic-*co*-glycolic acid) (mPEG–PLGA), to improve the efficiency of ofloxacin delivery at the site of action and inhibition of its extrusion. Since polyethylene glycol (PEG) is commonly used for drug conjugation and has the ability to bind DNA [31] and block drug efflux pump [32], the hypothesis was that mPEG–PLGA will improve antibacterial activity. The copolymer methoxy poly(ethylene glycol)-b-poly(lactic-*co*-glycolic acid) (mPEG–PLGA) was prepared by ring-opening polymerization of PLGA and mPEG in the presence of stannous octanoate as a catalyst. Ofloxacin encapsulated mPEG–PLGA and PLGA nanoparticles wa prepared by the emulsion solvent evaporation method. The nanoparticles exhibited a smooth spherical shape and were heterogeneous in their size; no aggregation or adhesion was observed. The obtained nanoparticles were tested on clinically important human pathogenic strains (*E. coli*, *P. aeruginosa*, *Proteus vulgaris*, *Salmonella typhimurium*, *Klebsiella pneumoniae*, and *S. aureus*) and markedly improved bacterial uptake and bacteriocidal activity compared to free ofloxacin. The ofloxacin–mPEG–PLGA nanoparticles displayed higher antibacterial activity, efficient bacterial uptake, sustained release, and strict control of bacterial growth. PEGylation increased bacterial membrane permeability, allowing the accumulation of mPEG–PLGA nanoparticles inside the cells to a greater extent than PLGA nanoparticles. The nanoformulation also delayed the development of bacterial resistance in comparison with the free drug [30].

Pure nanodrugs (PNDs) are forms of carrier-free therapeutic agents, i.e., nanoparticles, which are composed entirely of pure drug molecules [33]. Xie and coworkers designed propeller-shaped ciprofloxacin and norfloxacin PNDs which could form nanosized aggregates. The aim of the study was to obtain compounds that can be used as both therapeutic drugs and imaging agents by aggregation-induced emission (AIE) technique. The emission of AIE-active luminogens is poor in solution and increases upon their aggregation in response to restricted molecular motions. Such nanoaggregates are frequently obtained using propeller-shaped molecules [34]. The drug derivatives were synthesized from fluoroquinolone, perfluoroaryl azide, and an aldehyde in acetone solution (Scheme 2). Compounds **8a**–**8d** and **8g** behaved as AIE-active luminogens showing fluorescent properties with the quantum yield up to 11% and formed nanoaggregates with the size ranging from 39 to 127 nm. These forms were used as luminescent dots to image bacterial cells and exhibited an increase of antibacterial activity against *E. coli*, probably due to their higher local concentration or enhanced uptake [35].

**Figure:**
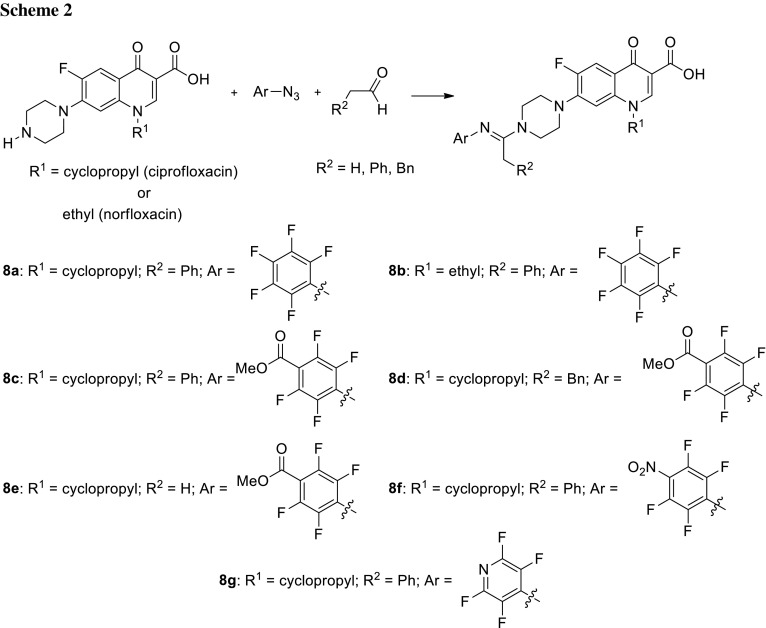

## Polymers

Polymer antibiotic conjugates afford lower toxicity, increased solubility, and prolonged activity of the drug, which have extensive applications in many fields, such as food packing or medical items [36]. They show remarkable high activity against the resilient biofilms [37]. Localized delivery methods based on physical stabilization of antibiotics in a polymer matrix such as a hydrogel or self-eluting polymer can release chemotherapeutics at the target region to maintain a high local concentration without exceeding systemic toxicity limits [38].

Gelatin is a water-soluble functional protein obtained by partial hydrolysis of collagen, widely employed in biomedical (tissue engineering) and food science. Especially in pharmaceutical field is commonly used for the preparation of drug delivery system (e.g., capsules, tablets, and emulsions) [39]. Cirillo and coworkers performed synthesis of biomacromolecules based on gelatin with anti-microbial properties of fluoroquinolone-type synthetic antibiotics [40]. Covalent linkage of the antibiotic was carried relatively simple by a radical process without the use of organic solvents, under mild reaction conditions, involving the residues in the side chains of gelatin able to undergo oxidative modifications. Ciprofloxacin, levofloxacin, and lomefloxacin were conjugated to gelatin in the presence of water-soluble redox initiators able to generate free radical species at room temperature under an inert atmosphere (Scheme 3). The synthetic strategy involved application of the ascorbic acid/hydrogen peroxide redox pair as radical initiators. Biocompatibility was tested on hBM–MSCs cell lines and all the samples were found to be non-toxic and well tolerated. No significant reduction in the cell viability was recorded after incubation with the anti-microbial conjugates up to concentration of 2 mg/cm^3^. Bioactive polymers were investigated against *K. pneumoniae* and *E. coli*. Biomacromolecules were able to inhibit growth of pathogen species; however, only ciprofloxacin conjugate showed the same minimal inhibitory concentration (MIC) values in comparison with the free drug, while for levofloxacin and lomefloxacin conjugates, lower antibacterial activities were recorded with respect to the corresponding parent drugs [40].

**Figure:**
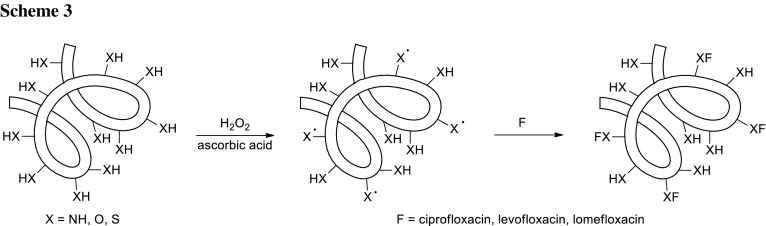

Poly(2-oxazoline)s (POx) are also non-toxic polymers with adjustable hydrophilicity and easily modified end-groups [41]. The antibiotic ciprofloxacin was covalently attached to the chain of poly(2-methyloxazoline) (PMOx), poly(2-ethyloxazoline) (PEtOx), and PEG (Scheme 4) [42]. Anti-microbial activity of the novel conjugates was tested against *S. aureus*, *Streptococcus mutans*, *E. coli*, *P. aeruginosa,* and *K. pneumoniae*. The direct coupling of PMOx and ciprofloxacin (compound **9a**) resulted in drastically low biological activity. It could be caused by reduced affinity to an enzyme or lowered diffusion ability into the bacterial cell; thus, alternative conjugates having a spacer between antibiotic and the polymer were prepared. The conjugates with spacer (**9b**) exhibited molar MIC values for some strains (e.g., *S. aureus*) lower than the pristine drug, while the activity was linearly increasing with shorter PMOx chain lengths. Conjugation of ciprofloxacin and quaternary ammonium compound via PMOx did not result in higher activity. The conjugates prepared with PEtOx as well as PEG (**9c**) revealed a strong activity dependence of the conjugate type, increasing in the order PEG > PEtOx > PMOx. The hemocompatibility of the prepared polymers was explored and HC_50_ (hemolytic concentration at with 50% blood cells is lysed) was determined with use of porcine blood cells. All values were above 5000 µg/cm^3^ indicating low hemotoxicity of the conjugates obtained [42].

**Figure:**
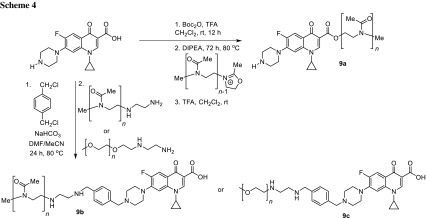

**Fig. 3:**
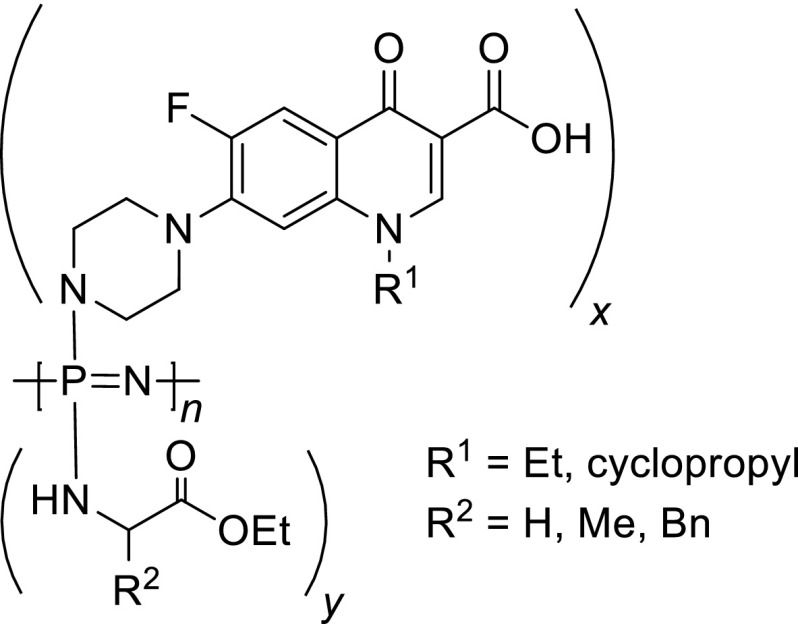
Structures of polyphosphazene–fluoroquinolone polymers. R^1^ = Et for norfloxacin and cyclopropyl for ciprofloxacin, R^2^ = H, Me, Bn for glycine, alanine, or phenylalanine, respectively

Polyphosphazenes are hybrid polymers with an inorganic backbone of alternating phosphorus and nitrogen atoms with two side groups attached to each phosphorus. Hydrolytically sensitive polyphosphazenes are formed when amino-acid ethyl ester groups are linked to the polymer backbone via the amino terminus [43]. The products of hydrolysis are non-toxic and contain parent amino acids, ethanol, phosphates, and ammonia, a mixture that results in a near-neutral pH [44]. Tian and coworkers prepared polyphosphazenes containing amino-acid esters (glycine, alanine, and phenylalanine) and ciprofloxacin or norfloxacin linked by piperazinyl group (Fig. 3). The polymers containing 12–25 mol% antibiotics and 75–88 mol% amino-acid esters were synthesized by macromolecular substitution using allyl protected carboxyl group of antibiotic, followed by the removal of allyl group under mild condition. Nano/microfibers of selected antibiotics were prepared by electrospinning technique. Hydrolysis behavior over a 6-week period was studied using different polymers as films and as nano/microfiber mats for in vitro experiments based on their mass lost and the pH of the hydrolysis media. All polymers were sensitive to hydrolysis. The degradation speed was dependent on the amino-acid esters attached to a polymer backbone and followed a trend glycine > alanine > phenylalanine. The bulkier substituents more effectively shielded the polyphosphazene backbone from access to water. After the 6-week study, about 87 and 82% of polymers were left as films for alanine and glycine ciprofloxacin conjugates. In vitro antibacterial tests performed against *E. coli* demonstrated antibacterial capabilities as long as the antibiotic was being released [45].

He and coworkers synthesized copolymers containing monomers of methacrylate with ciprofloxacin, quaternary ammonium salts (QAS), and butyl acrylate by free radical copolymerization (Scheme 5). QAS were incorporated into polymers to increase water solubility as well as to improve the anti-microbial activities. These antibacterial agents exhibited excellent cell membrane penetration properties [46]. When positively charged, QAS adsorb onto the negatively charged bacterial cell by electrostatic interaction surface, diffuse through the cell wall, disrupt plasma membrane, and lead to bacterial death by the release of the cytoplasmic contents [47]. Polymerization was performed in ethanol at 65 °C for 24 h using azobisisobutyronitrile as an inhibitor. The molecular weight of the copolymers was ranging from 10,000 to 15,000. Anti-microbial activity was tested against *E. coli* by means of zone inhibition method. Bacterial growth was inhibited which indicated excellent antibacterial properties. The highest antibacterial activity was obtained for the copolymer **10** which consisted of 56.4, 4.3, and 39.3 mol% monomers of QAS (*x*), ciprofloxacin (*z*), and butyl acrylate (*y*), respectively. MIC value determined by serial dilution method against *E. coli* reached 4.0 ppm. Hydrophobicity increase by incorporation of more butyl side chains enhanced biological activity; however, excessive hydrophobicity caused aggregation and precipitation in water. The morphology of bacteria 10 min after treatment with 50 ppm of **10** was characterized by confocal laser scanning microscope and showed bacterial membrane damage, as well as bacterial components leakage [48].

**Figure:**
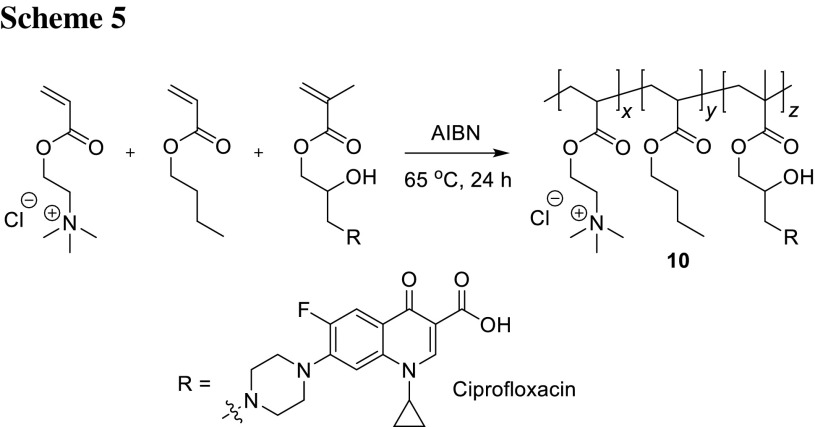

Prodrugs are molecules that contain drug pharmacophore and specialized non-toxic protective groups utilized in a transient manner to alter or eliminate the undesirable properties of the parent drug molecule. They allow to release of the drug moiety in the site of action and thus exploit localized activity of free drug molecule. Sobczak and coworkers synthesized polyester prodrugs of norfloxacin based on two-, three- and four-arm, star-shaped oligoesters: poly(ε-caprolactone) (**11a**), poly(d,l-lactide) (**11b**), and the copolymer of these homopolymers. The polymerization reactions were performed via ring-opening of cyclic esters in the presence of stannous octoate as a catalyst and poly(ethylene glycol) (*m* = 2), glycerol (*m* = 3), or penthaerythritol (*m* = 4) as initiators. The reaction yields were in the 44–100% range and the determined average molecular weights were assessed between 2900 and 9600 Da. The oligomers were subsequently reacted with fluoroquinolone antibiotic (Scheme 6). Authors suggest that these polymers are potential candidates to be applied as drug delivery carriers [49].

**Figure:**
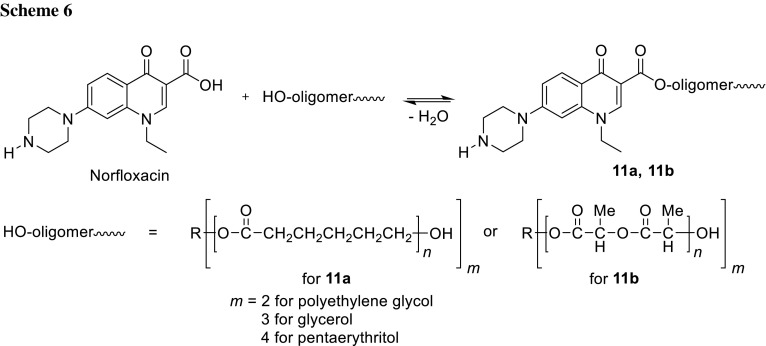

Polysaccharides can serve as polymers for prodrugs’ formation of delayed or targeted delivery [50]. The cellulose ethers hydroxypropylcellulose (HPC) and hydroxyethylcellulose (HEC) were used by Hussain research group to obtain macromolecular prodrugs of moxifloxacin and ofloxacin. The carboxyl groups of the antibiotics were activated by *p*-toluenesulfonyl chloride and esterification was performed in the presence of trimethylamine (Scheme 7). The products of esterification were soluble in water and organic solvents. The degree of substitution was high; the polymers contained 21–29 mg of moxifloxacin or 32–42 mg of ofloxacin per 100 mg of conjugate, respectively, which make them useful for tablets production with acceptable size (500–1000 mg). Moxifloxacin–HPC conjugate self-assembled into nanowires (diameter approximately 30 nm), while one of the moxifloxacin–HEC conjugates formed nanoparticles with diameters ranging from 150 to 350 nm. Nanoparticles of ofloxacin were obtained in the size range 100–250 and 150–210 nm for HPC and HEC conjugates, respectively. Pharmacokinetic studies were performed using a rabbit model upon oral administration. Both the conjugated polymers were able to hydrolyze and the release was highly delayed enhancing antibiotics plasma half-life, for moxifloxacin over 24 h and for HPC and HEC conjugates of ofloxacin 18.07 and 20.71 h, respectively. These values are close to once daily dosing ideal value. Drug release tests of the moxifloxacin conjugates were performed in simulated gastric and intestinal fluids at 37 °C. Hydrolysis occured faster at pH 7.4 than 1.2 which makes these prodrugs interesting for targeted delivery to the colon and distal small intestine [51, 52].

**Figure:**
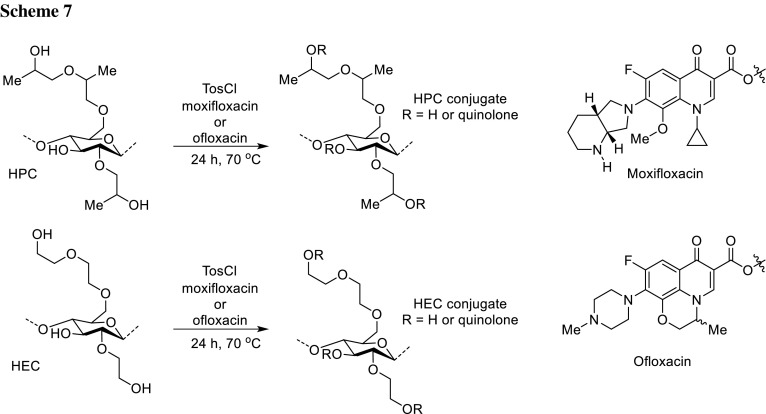

## Proteins and peptides

Kumar and coworkers synthesized enrofloxacin conjugated with bovine serum albumin (BSA) to use the conjugate as an antigen capable of producing polyclonal antibodies against the antibiotic. Enrofloxacin belongs to antibacterials commonly used in veterinary practice in the treatment of infectious diseases as well as prophylactic agent; therefore, the produced antibodies could be employed for the detection of antibiotics in milk samples. To obtain immunogens, the carbodiimide reaction was employed with 1-ethyl-3-(3-dimethylaminopropyl)carbodiimide (EDCI) as a crosslinker. Polyclonal antibodies were successfully produced in rats, which were confirmed by indirect ELISA [53].

German and coworkers prepared conjugates of ciprofloxacin and ofloxacin with dipeptides or bisarylurea to expand action of the antibiotics on the substrate-based inhibitors of bacterial efflux pumps. Fluoroquinolone resistance in *S. aureus* may be caused by the *norA*-encoded and *mepA*-encoded fluoroquinolone efflux pump systems [54]; therefore, coadministration of bacterial efflux pump inhibitors with antibiotic agents led to overcome the efflux-mediated resistance [55]. Bisaryl urea and dipeptide components, known inhibitors of NorA and MexAB pumps, respectively, were selected for incorporation to the C7 position of fluoroquinolone core. The conjugation of urea was achieved by attachment of bisaryl urea to the C7 piperazine of ciprofloxacin or C7 amine of ofloxacin precursor in direct alkylation (**12a**, **12b**) (Scheme 8). Ciprofloxacin conjugates bearing Phe–Lys or Lys–Phe moiety was obtained with use of standard amino-acid coupling chemistry to modify the C7 piperazine moiety (**13a**, **13b**) (Scheme 8). The novel compounds were tested against *E. coli*, *P. aeruginosa*, and *S. aureus* strains. In all cases, activities of conjugates were significantly lower than the parent drugs. None of the conjugates achieved appreciable inhibition of efflux pump system at any tested concentration in *P. aeruginosa* efflux inhibition studies. However, ofloxacin–urea conjugate **12b** exhibited the highest inhibitor potencies of NorA and MepA efflux pump systems in *S. aureus* efflux inhibition assays and at 0.5 µM concentration inhibited NorA-mediated and MepA-mediated efflux by 73.6 and 53.4%, respectively [56].

**Fig. 4:**
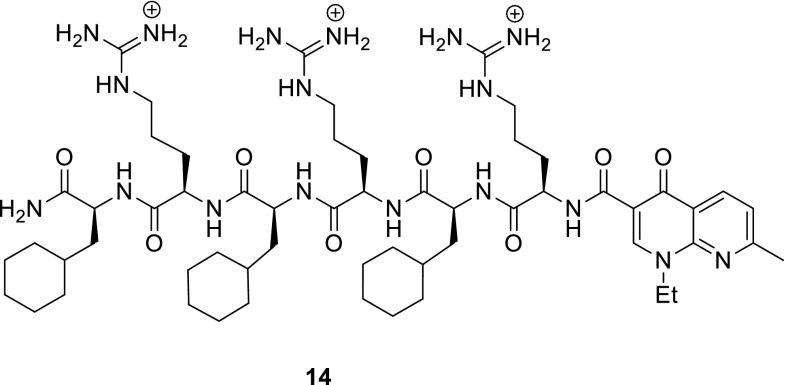
Structure of peptide–quinolone conjugate **14**

Ahmed and Kelley designed conjugates of nalidixic acid and small peptides (3–12 amino acids) containing cationic and hydrophobic amino-acid residues to improve cellular uptake. Oligopeptides bearing positive charge exhibit affinity to negatively charged phosphodiester anions of DNA allowing for accumulation of the drug at the fluoroquinolone site of action [57]. The novel compounds were prepared by solid-phase peptide synthesis by incorporation of hydrophobic cycohexylalanine and positively charged d-arginine. Subsequently, nalidixic acid was conjugated to peptide scaffolds by carbodiimide chemistry. The conjugates were tested against *S. aureus* MRSA and MSSA strains. The most hydrophobic compounds carrying a net + 3 molecular charge were found to be highly active in both strains of the bacteria and exhibited the highest potency as DNA gyrase inhibitors by attenuating replication levels. Compound **14** was evaluated for membrane disruption properties and the results indicated that it does not alter membrane perturbation. Toxicity of **14** was tested in two types of human fibroblasts and the IC_50_ values were more than tenfold higher for the fibroblasts vs. the *S. aureus* strains tested. This trend indicates that the antibacterial agent **14** possess a suitable therapeutic window [58] (Fig. 4).

Riahifard and coworkers prepared conjugates of anti-microbial cationic peptides with fluoroquinolones. They conjugated amphiphilic linear or cyclic peptides bearing arginine and tryptophan residues with levofloxacin or levofloxacin-Q to enhance their ability to penetrate through bacterial lipopolysaccharides (Scheme 9). The compounds were synthesized using Fmoc/*tert*-Bu solid-phase peptide synthesis and tested against *K. pneumoniae* and *S. aureus* MRSA strains. The conjugate **15b** demonstrated higher antibacterial activity than the parent drug. Other compounds exhibited reduced activity and no synergistic antibacterial effect, probably due to the incomplete hydrolysis of the conjugate [59] (Scheme 9).

**Figure:**
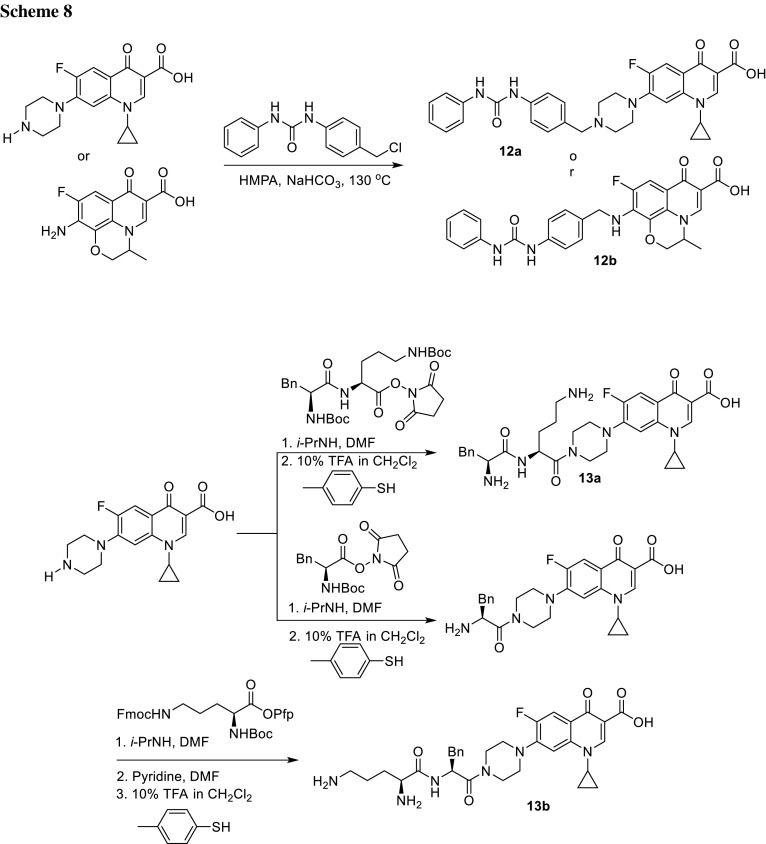

**Figure:**
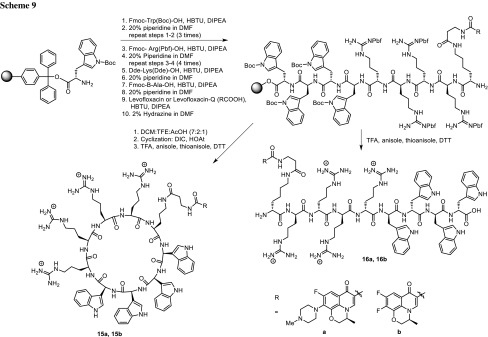

**Fig. 5:**
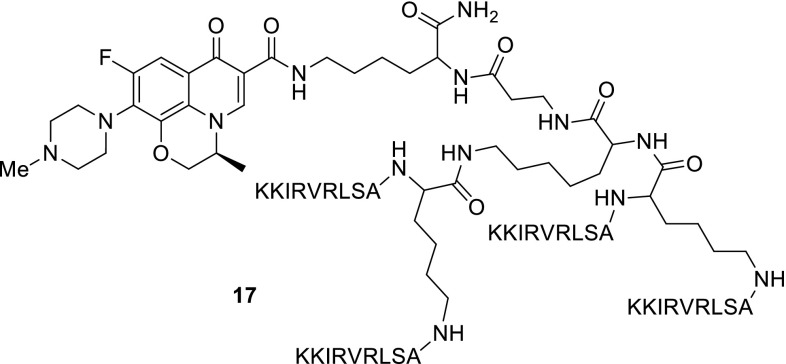
Structure of peptide M33-levofloxacin hybrid **17**

Another research group, Ceccherini and coworkers, employed solid-phase peptide synthesis to conjugate carboxylic group of levofloxacin with an amine group of lysine side chain in the M33 peptide. M33 is a tetra-branched peptide with high activity against Gram-negative bacteria currently under preclinical development [60]. Antibacterial activity of the obtained conjugate **17** was tested in anti-microbial assays against *P. aeruginosa* and *E. coli*; however, the results indicated that the conjugation did not induce enhanced antibacterial properties [61] (Fig. 5).

Next example of cationic anti-microbial peptide conjugated with fluoroquinolone consists of levofloxacin modified with the Pep-4 peptide, which is based on human beta defensin-3 of RGRRSSRRKK-NH_2_ sequence. The incorporation of antibiotic was performed by covalent modification of levofloxacin carboxyl moiety (preactivated to an acyl fluoride) to three primary amino groups present in the peptide (two lysine side chains and N-terminus) via direct acylation (Scheme 10). The antibacterial properties of the obtained conjugate **18** were evaluated against Gram-positive bacterium *Bacillus cereus* and Gram-negative *E. coli*. The antibacterial assays were conducted at three different ionic strengths, because the effectiveness of the anti-microbial peptides may be limited under salt conditions consistent with physiologically relevant environments. The conjugate exhibited substantially better activity in comparison with the free peptide at higher ionic strengths. Depolarization studies indicated that the conjugate was able to disrupt membrane integrity in *E. coli* to a greater degree than the free peptide possibly due to its higher hydrophobicity (log*D* of conjugate measured in 10 mM phosphate buffer pH 7.4 and 1-octanol was − 1.65, while the non-conjugated peptide demonstrated log*D* of − 2.57). Moreover, the findings suggested that enhanced antibacterial potency is not caused by the extracellular release of the free drug, since coadministration of unmodified Pep-4 with free levofloxacin resulted in significantly lower activity than in case of the conjugate [62].

**Figure:**
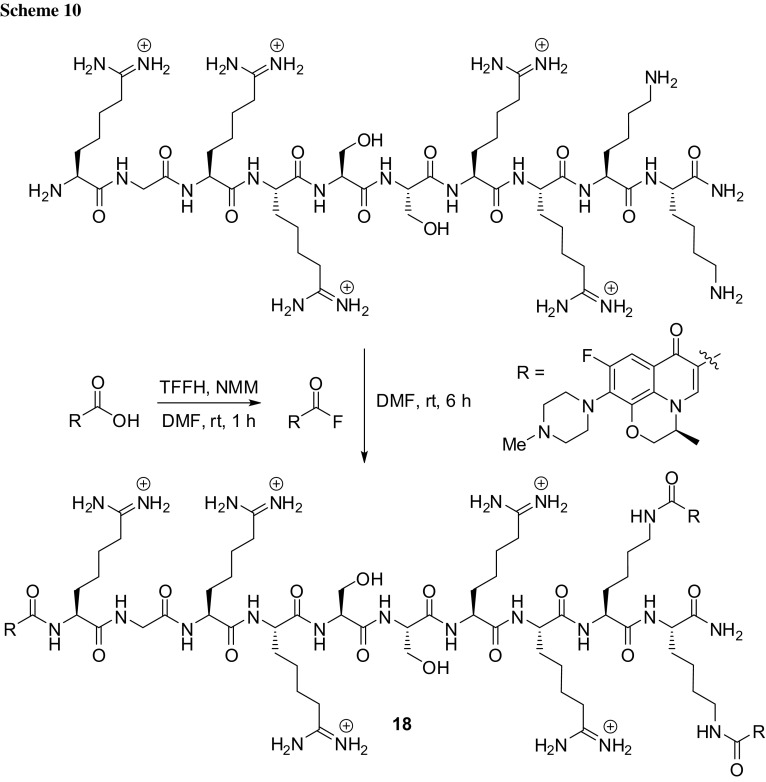

**Fig. 6:**
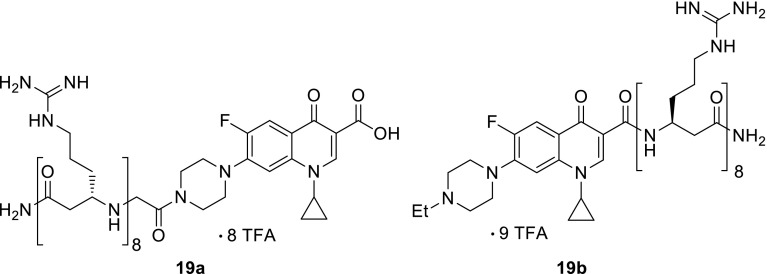
Structures of fluoroquinolone-β-octaarginine conjugates **19a**, **19b**

Other research group designed enrofloxacin and ciprofloxacin derivatives of β-octaarginine, polycationic cell-penetrating peptide non-metabolized, and stable against proteases. The peptide scaffold was attached at the piperazine amino and at the carboxylic acid groups of ciprofloxacin (**19a**) and enrofloxacin (**19b**), respectively, to create amide bonds resistant to enzymatic cleavage. Evaluation of antibacterial properties was performed on a panel of 20 aerobic Gram-positive and Gram-negative bacterial strains; however, none of the obtained conjugates exhibited enhanced anti-microbial activity with reference to parent drugs [63] (Fig. 6).

## NO donors and analogs

Nitric oxide (NO) is an inorganic free radical gaseous molecule important in a variety biofilm-forming species for signaling. Used at low, sub-lethal concentrations, NO is capable to induce a transition from the sessile biofilm state to a dispersed (planktonic) mode of growth [64]. Due to a short half-life of NO (0.1–5 s) and its extreme chemical reactivity, NO-donor molecules are used to deliver the drug into systems, where biofilms are prevalent [65].

Benzofuroxans are stable in the air compounds able to generate external NO. They find applications as vasodilators and exhibit antianginal properties [66]. Chugunova and coworkers synthesized benzofuroxan salts **20**–**22** with several fluoroquinolones, namely, sparfloxacin (**a**), ciprofloxacin (**b**), norfloxacin (**c**), and lomefloxacin (**d**), formed by hydrolysis of benzofuroxans (Scheme 11). The bacteriostatic and bacteriocidal activity of obtained salts was tested for anti-microbial efficacies in Gram-positive (*S. aureus* and *B. cereus*) and Gram-negative (*P. aeruginosa* and *E. coli*) bacterial strains. The compound **20d** showed the best antibacterial activity, even eight times higher than original drug lomefloxacin. Moreover, the tested compounds exhibited very weak toxicity to human blood cells—hemolysis did not exceed 1% in concentrations 0.19–3.9 mg/dm^3^ [67].

**Figure:**
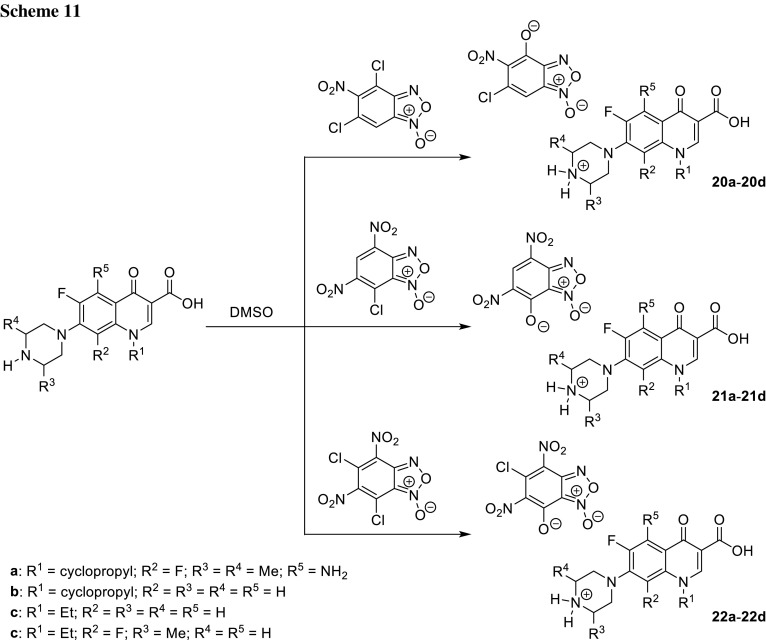

**Figure:**
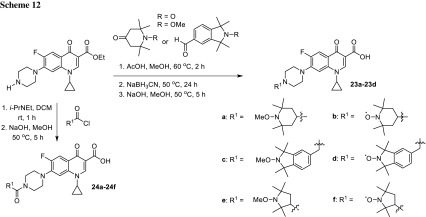

Nitroxides are also useful crystalline solids structurally similar to NO. They undergo redox chemistry and exhibit antibacterial effect. Ciprofloxacin–nitroxide hybrids **23b**, **24b**, **23d**, **24d**, and **24f** were synthesized and evaluated as anti-biofilm agents (Scheme 12). The methoxyamine derivatives **23a**, **24a**, **23c**, **24c**, and **24e** were prepared as a control to enable direct comparison (Scheme 12). Compounds **23a**–**23d** were obtained via a tertiary amine linker by reductive amination followed by deprotection of ethyl ciprofloxacin esters, while compounds **24a**–**24f** were synthesized using amide bond coupling with corresponding acyl chloride. The desired products were obtained in good-to-excellent yields (64–98%) and antibacterial activity was measured against biofilm-forming *P. aeruginosa* strain. The results indicate that the nitroxide hybrids possess dual-action effect. The most active hybrid **23b** showed dispersal activity towards mature biofilm and antibiotic action by means of eradication of the newly dispersed bacteria up to 95% at 40 µM [68]. Compounds **24b**, **24d**, and **24f** also displayed good anti-biofilm activity. Compound **24d** removed 85% of existing biofilms at 20 µM (10.95 µg/cm^3^). Free ciprofloxacin was ineffective at biofilm removal; however, the addition of nitroxide moiety to the piperazine ring through amide bonds, in general, has resulted in decreased activity against planktonic forms of bacteria. Selected compounds examined in human muscle rhabdomyosarcoma and human embryonic kidney 293 (HEK-293) cells were found to be non-toxic up to the highest concentrations used (40 µM) [69].

## Anionic compounds

Chronic lung infections are caused by accumulation mucus lining the airway of the lungs, where Gram-negative aerobes are known to evade host defenses. *P. aeruginosa* is one of the common pathogens with an ability to form biofilm and colonize pulmonary tract. Long and coworkers hypothesized that negatively charged compounds bearing sulfoxy or carboxy groups could serve as inhibitors of these biofilm-producing strains and penetrate the alginate component of *P. aeruginosa* extracellular polymeric substance. To evaluate this hypothesis, they designed anionic fluoroquinolones and tested their pseudomonal inhibition efficiency against non-mucoid and mucoid strains (*P. aeruginosa* PAO1 and PAO581, respectively) by determining zones of inhibition, MIC, and MBC (minimal bactericidal concentration). Compounds **25a**–**25c** were prepared from ciprofloxacin and the appropriate cyclic anhydrides in DMSO, while hybrids **25d** and **25e** were obtained by alkylation of the piperazinyl ring with bromides of corresponding methyl esters followed by acid hydrolysis (Scheme 13). The modifications resulted in decrease of the antibacterial activity. The most active compound **25c** was found to be inferior compared to the lead compound, ciprofloxacin. The data suggest that novel compounds penetrate biofilm less efficiently than standard antibiotics [70].

**Figure:**
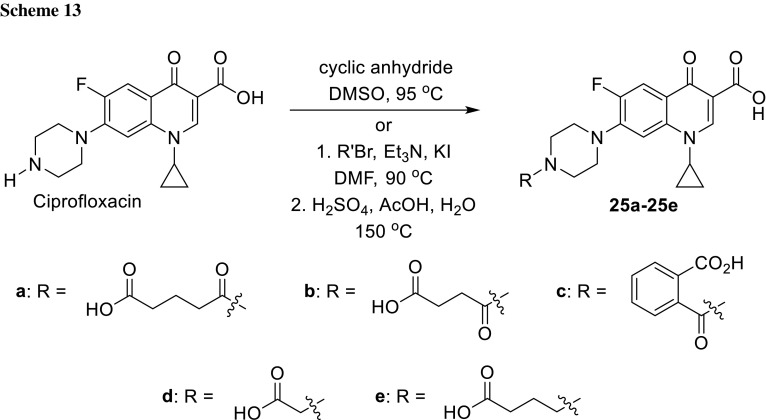

## Siderophores

Certain pathogenic microorganisms under iron-limited conditions synthesize and excrete low-molecular-weight molecules called siderophores, able to chelate low-bioavailable Fe(III) from the surrounding environment and compete with the host for this element [71]. Siderophore–Fe(III) complex is recognized by the dedicated membrane receptors and transported into the bacterial cell. Then iron is released from the complex for further use, which allows the bacteria to survive in iron-deficient media. Sideromycins are natural conjugates of an antibiotic molecule and a siderophore analog, often connected by a hydrolyzable linker that can be cleaved by endogenous enzymes. These components are recognized and transported into the targeted bacteria by the siderophore-dependent iron uptake pathways. After the sideromycin has been transferred across the bacterial envelope, the antibiotic is released [72]. This natural strategy can be used in Trojan horse approaches using synthetic siderophores as vectors to transport antibiotics into the bacterial cells [73].

Although citrate has relatively low affinity to Fe(III) [74], it is used by *E. coli* as an exogenous siderophore [75]. Md-Saleh and coworkers prepared conjugates of ciprofloxacin with a monocitrate unit linked via stable amide bond on the piperazinyl ring. Methanoate ciprofloxacin esters were subjected to the reaction with citrates by EDCI-mediated coupling, then deprotected furnishing conjugates **26a** and **26b** in good yields (Scheme 14). Anti-microbial activity of the obtained compounds was tested against several common pathogens, inter alia *S. aureus*, *Staphylococcus epidermidis*, *P. aeruginosa*, *Serratia marcescens*, *Burkholderia cepacia*, and *E. coli.* The inhibition activity for both novel compounds was comparable to the clinic drug ciprofloxacin and, however, slightly lower for the majority of the strains tested. Compound **26b** has been subjected to additional tests to explore its cell membrane permeability; however, the data showed that there was no additional uptake via an iron–citrate pathway and the conjugate was not recognized by Fec system [76].

**Figure:**
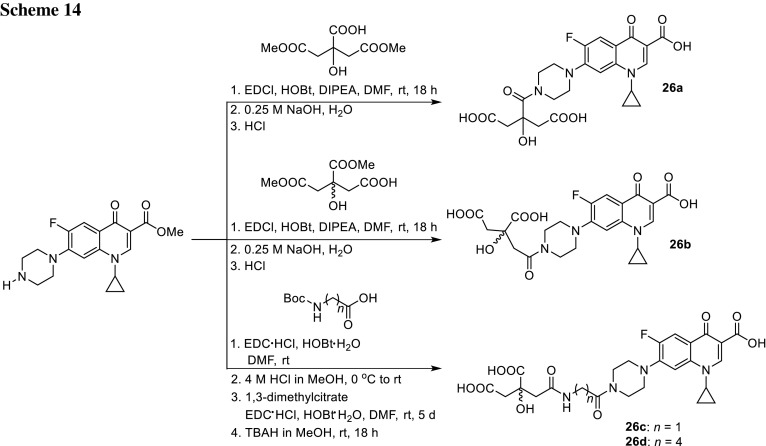

**Fig. 7:**
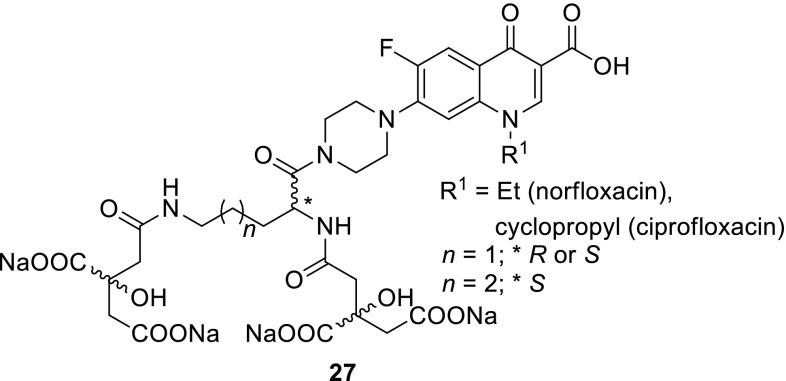
Structures of siderophore and fluoroquinolone hybrids **27**

Milner and coworkers continued the study and synthesized analogical conjugates with longer linkers between siderophore and ciprofloxacin molecules **26c** and **26d** (Scheme 14). The modification resulted in decrease of antibacterial action as well as gyrase inhibitory activity. They designed also staphylococci-targeted citric acid–ciprofloxacin or norfloxacin conjugates based on staphyloferrin A, siderophore that is secreted by *S. aureus*. This siderophore is the most efficient under slightly acidic conditions. Its optimum pH lies close to that found for the average skin (5.5). Therefore, novel compounds could be employed in skin infection treatment. Moreover, this type of modification could improve water solubility of the conjugates. Compounds **27** were screened against a collection of reference and clinical isolates associated with infections in humans. They exhibited reduced activity and were less effective at inhibiting DNA gyrase than ciprofloxacin on its own, probably due to electrostatic repulsion or steric clashes of the modified drug when interacting with its binding site in the enzyme [77, 78] (Fig. 7).

Pyochelin is a siderophore recognized by FptA receptor common to several pathogenic *Pseudomonas* and *Burkholderia* species, Gram-negative bacteria causing severe and lethal lung infections especially for immunocompromised patients or subjects with cystic fibrosis [79]. Mislin research group synthesized pyochelin–fluoroquinolone conjugates using various types of linkers for norfloxacin or ciprofloxacin (Scheme 15). The adducts were tested against *P. aeruginosa* strains: wild-type, pyochelin-deficient, and TonB-deficient (TonB is a key protein involved in the iron assimilation process). Labile-arm conjugates **28b**, **28d**, **28f**, **28h** showed lethal activity; however, only for compounds **28b**, **28d,** the effects were as pronounced as for free norfloxacin. Compounds **28f**, **28h** were less active, presumably due to their poor water solubility [80, 81].

**Figure:**
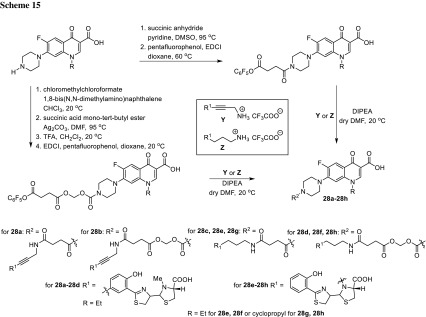

Enterobactin is a tricatecholate siderophore secreted by *Escherichia*, *Salmonella*, and *Klebsiella* species [82]. Zheng and coworkers obtained ciprofloxacin–enterobactin conjugates in the synthetic route, as presented in Scheme 16. The conjugate **29a** was found to be recognized by transport system proteins and successfully delivered to the cytoplasm of *P. aeruginosa* as well as *E. coli* causing growth inhibition of these microbes [83]. The conjugates **29b**, **29c** having labile (alkoxy)alkyl ethers linkers were found to be hydrolyzable in the hydrolytic stability tests; however, in anti-microbial activity assays performed for *E. coli* strains, their activity was attenuated by tenfold (MIC of 1 µM) relative to ciprofloxacin. The modest growth inhibitory activity was probably caused by the release of unmodified ciprofloxacin in the growth medium rather than by targeted delivery [84].

**Figure:**
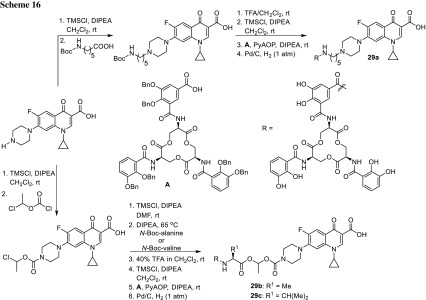

**Fig. 8:**
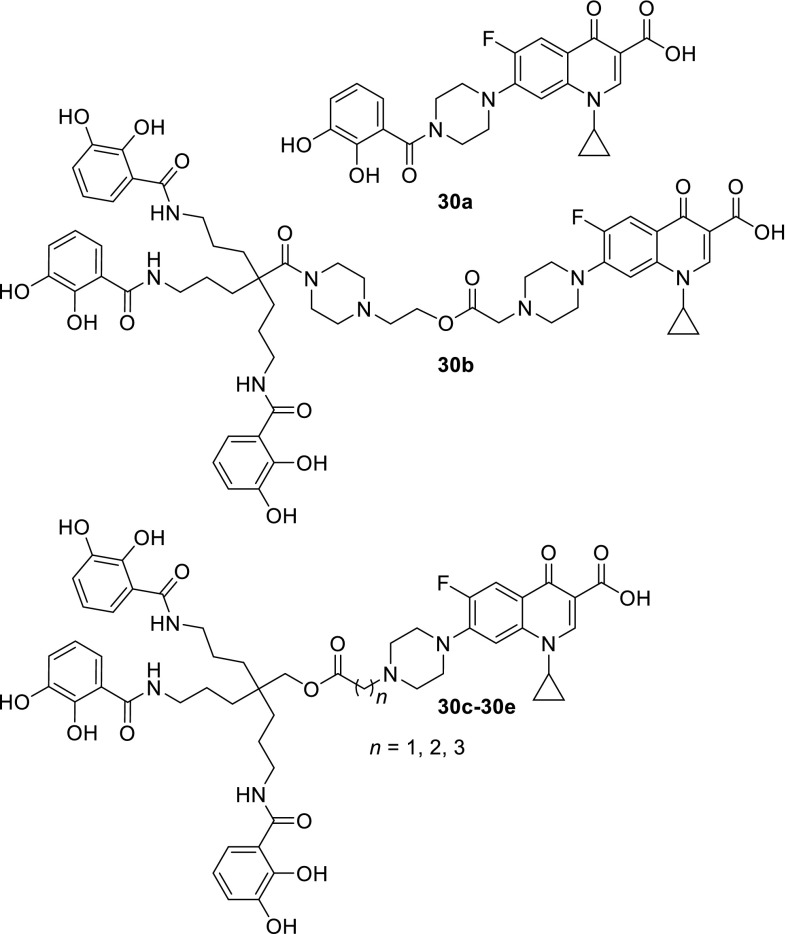
Structures of catecholate–ciprofloxacin conjugates **30a**–**30e**

Catecholate–ciprofloxacin conjugates (Fig. 8) were also synthesized by Fardeau and coworkers and tested against *P. aeruginosa* strains. The antibacterial activities of the hybrids were moderate in both iron-rich and iron-deficient media and inferior to ciprofloxacin. This could be related to low solubility in aqueous media and/or the absence of hydrolysis of the hybrids. The hemolytic activity of the conjugates was low which indicated low cytotoxicity of obtained compounds [85].

**Fig. 9:**
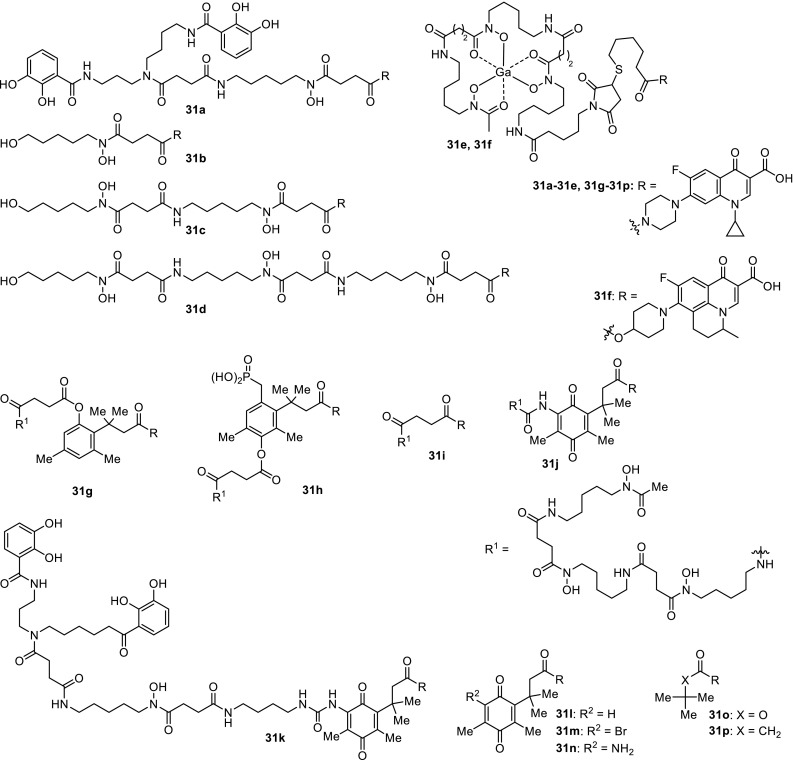
Structures of fluoroquinolone–siderophore hybrids **31a**–**31k**

Miller’s research group designed and prepared a series of sideromycins, which were evaluated for their antibacterial properties against *Enterococcus faecium*, *S. aureus*, *K. pneumoniae*, *A. baumannii*, *P. aeruginosa*, *Enterobacter aerogenes*, and *E. coli* bacterial strains. Biscatecholate–ciprofloxacin conjugate **31a** showed no antibacterial activity against all tested bacteria, whereas the parent ciprofloxacin was highly active [86]. Mono-, bis-, and trihydroxymate derivatives of ciprofloxacin **31b**–**31d** were synthesized based on the structure of desferrioxamine B, trihydroxymate siderophore produced by several species of *Nocardia*, *Streptomyces*, *Micromonospora*, *Arthrobacter*, *Chromobacterium*, and *Pseudomonas* [87]. This conjugates showed reduced spectrum of activity relative to the broadly active parent antibiotic. These compounds were subjected to further experiments to determine if they were actively transported into the bacterial cells. Compound **31d** was found to enter *S. aureus* cell membrane via protein-mediated siderophore-uptake pathways [88]. Compounds **31e**, **31f** were synthesized using the thiol-maleimide strategy from desferrioxamine B and fluoroquinolone derivatives, ciprofloxacin and nadifloxacin, respectively. The conjugates featured Ga(III) as a chelator. *Mycobacterium smegmatis* and *E. coli* were not affected by these compounds; however, conjugate **31f** acted as strong inhibitor of *Bacillus subtilis* growth [89]. Compounds **31g**, **31h** were designed to enhance antibacterial activity of prodrugs by ensuring the intracellular release of antibiotic from ciprofloxacin–desferrioxamine B conjugate. Biologically active components of the hybrids were joined with the use of esterase and phosphatase triggered drug release linkers containing ‘trimethyl lock’. The chemical structure of ‘trimethyl lock’ is an *O*-hydroxycinnamic acid which unfavorable steric interactions between the three methyl groups encourage rapid spontaneous lactonization to form a hydrocoumarin after enzymatic hydrolysis [90]. The compound **31i** possessing a succinyl linkage, stable under physiological conditions, was included in the biological studies as a control. The conjugates were evaluated for their ability to inhibit growth of *B. subtilis*, *S. aureus*, *P. aeruginosa*, *E. coli*, and *Micrococcus luteus* strains. The antibacterial activity of hybrid **31g** was moderate to good, although weaker than that of the ciprofloxacin [91]. Siderophore–ciprofloxacin conjugates with ‘trimethyl lock’ incorporating a urea linkage **31j**, **31k** were also prepared due to their appreciable stability and synthetic accessibility. Electrochemical and LC–MS studies revealed that the quinolone moiety in the linker was thermodynamically reducible and the expected lactonization was rapid. Complete release of the ciprofloxacin from the conjugate **31j** was achieved after 25 min under mild conditions (37 °C, 20-fold excess of sodium dithionite). Antibacterial activity assays indicated that drug release occurred inside the bacterial cells; however, the conjugates **31j**, **31k** were less active relative to the parent drug [92] (Fig. 9).

*N*-Acylated ciprofloxacin derivatives based on the ‘trimethyl lock’ without siderophore molecule **31l**–**31p** were also prepared and tested against *E. coli*, *P. aeruginosa*, *Mycobacterium vaccae*, *M. luteus*, *S. aureus*, *B. subtilis*, and *A. baumannii*. These compounds showed moderate-to-good activity against *M. vaccae* and Gram-negative pathogens, although inhibition was decreased in comparison with ciprofloxacin by 2–50-fold. The most active conjugate was found to be compound **31n** with MIC values against Gram-positive *S. aureus* and *M. luteus* superior to these determined for ciprofloxacin. This result suggests that compound **31n** may act through a dual-action mechanism presented in Scheme 17 by serving as a prodrug and covalent thiol-containing enzyme inhibitor [93].

**Figure:**
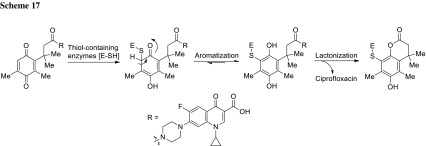

## Phosphonates

Osteomyelitis is an inflammatory process localized in bones often accompanied by bone necrosis resulting from an underlying microbial infection (caused primarily by *S. aureus*) [94, 95]. Difficulties in effectively treating this disease are consequence of physiochemical environment poorly accessible to the immune system. Therapy requires a large concentration of antibiotic to be maintained in infected bone over a long period of time; therefore, frequent intravenous administration of high drug doses is needed. Bisphosphonates are used in the medical praxis as anti-osteoporosis drugs due to their ability to adsorb to the hydroxyapatite, calcium phosphate bone mineral [96]. These strong metal ions chelators serve as targeting medicinal agents in bone diseases through rapid diffusion to osseous tissues in vivo [97]. Far’s research group prepared a series of osteotropic prodrugs for osteomyelitis prevention. The conjugates contained fluoroquinolone antibiotic and phosphonate moiety aimed at delivery the drug directly to the site of action. Moxifloxacin, gatifloxacin, and ciprofloxacin were used to produce prodrugs efficiently binding to bone tissue and able to release active fluoroquinolone molecules. The synthesized compounds included C3 aryl (**32a**), glycoamide (**32b**–**32h**), and thioglycoamide (**32i**) esters (Scheme 18) [98].

**Figure:**
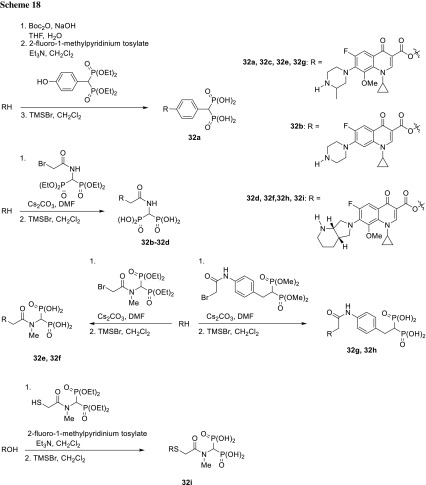

Furthermore, C7 hybrids have been prepared through addition of alkenes (**33a**, **33b**) or α,β-unsaturated carbonyl compound (**33c**) bearing bisphosphonate moiety to the amine group of fluoroquinolone. Similar phosphinyl **33d**–**33g** and methylenebisphosphonate **33h**, **33i** C7 conjugates were prepared [99]; however, only compounds **33c**, **33d** exhibited good inhibition activity against *S. aureus* (MIC values < 0.12 and 0.12 µg/cm^3^, respectively). Lower activity than the parent quinolones indicated that during 24 h assay, prodrugs were not able to release the parent drug. Binding to bone powder was at the very efficient level. The prepared compounds **32a**–**32d**, **32g**–**32i**, **33a**–**33c**, **33h**, and **33i** have been absorbed in 80–90% over 1 h, while conjugates **33d**–**33g** in 35–76%. Compounds **32b**–**32d** and **33c**–**33g** were proved to hydrolyze in plasma and release the free drug efficiently. Prodrugs **32b**–**32d** and **33c** did not require the participation of an enzyme to appreciably regenerate the parent fluoroquinolone in vitro. Prodrugs **32b**, **32c**, and **33c** tested in rats significantly reduced bacterial titer in the bone under exposure of 20.8, 15.8, and 17.3 mg/kg of body weight, respectively [97–99] (Scheme 19).

**Figure:**
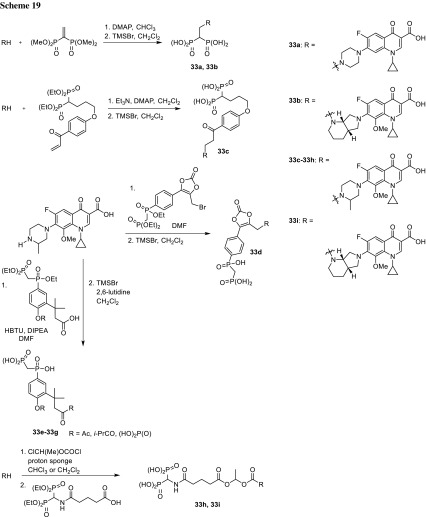

1-Hydroxybisphosphonates derivatives of ciprofloxacin (**34a**), gatifloxacin (**34b**), and moxifloxacin (**34c**) were synthesized with use copper(I) catalyzed azide-alkyne 1,3-dipolar cycloaddition reaction by McPherson III and coworkers (Scheme 20). Ciprofloxacin derivative **34a** possessed the highest antibacterial activity against a panel of clinically relevant bacteria including *B. subtilis*, *S. aureus*, *S. epidermidis*, *Enterococcus faecalis*, *E. coli*, *K. pneumoniae*, *P. vulgaris*, and *P. aeruginosa*. The osteotropic properties of the obtained compounds were evaluated using synthetic nanosized hydroxyapatite bone model. The adsorption level was in the range of 70–100% [100]. These hydroxybisphosphonate derivatives of fluoroquinolones can be considered as potential candidates for bone-targeted drugs.

**Figure:**
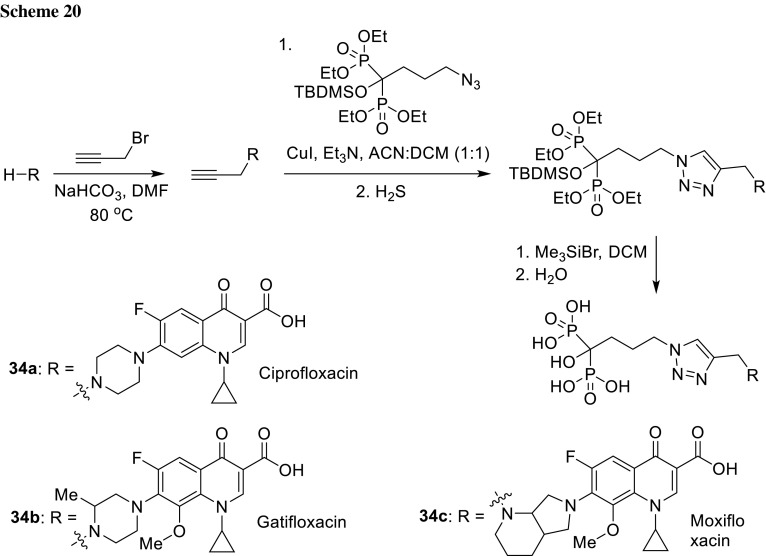

## Prodrugs with enhanced lipophilicity

Dubar and coworkers have proposed bioorganometallic strategy that led to improvement in antimalarial activity of fluoroquinolones. They designed ethyl esters of ciprofloxacin prodrugs bearing a ferrocenyl substituent at position N1 or C7 of the quinolone core **35a**–**35c**. The conjugates were obtained from fluorobenzoic acid transformed in two-step procedure into ethyl 3-(diethylamino)-2-(2,4,5-trifluorobenzoyl)acrylate (Scheme 21). The ester has been cyclized with the corresponding amines and subsequently reacted with ferrocenyl compounds. The products **35a**–**35c** were more active than ciprofloxacin against *Plasmodium falciparum*, malaria-causing parasite, possibly due to their enhanced lipophilicity. The drug molecule needs to penetrate multiple membranes present in the intracellular parasite to reach fluoroquinolone target, gyrase, or topoisomerase IV. Thus, the strong antiplasmodial effect was achieved as a result of hydrophobic capacity which facilitated transport of the drug across membranes. Toxicity of novel compound was tested in vitro using mouse spleen cells; however, therapeutic index was relatively low (selectivity index for the most active compound **35b** reached 8) [101]. Ferrocenyl derivatives seemed to be promising antimalarials, but considering their toxicity, they required further structure optimization. For this reason, the study was extended for conjugates **35d**, **35e** prepared in a similar way. The new conjugates were found to be dramatically more active than the parent drug not only against *P. falciparum* but also against tachyzoites of *Toxoplasma gondii* at very low concentrations. Toxicity was examined against mouse spleen cells and LLC-MK22 cell line. Cytotoxic effect was greatly reduced and therapeutic index for the adamantly derivative was above 60 and 100 for *T. gondii* and *P. falciparum*, respectively [102].

**Figure:**
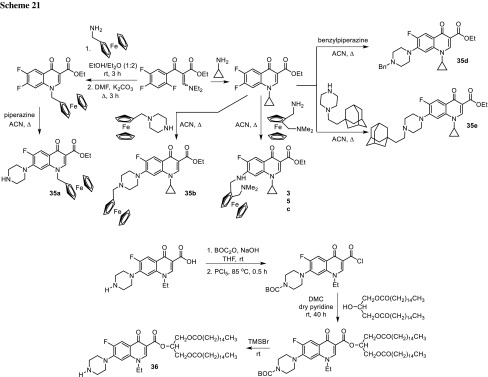

Dhaneshwar and coworkers employed ester prodrug strategy to improve oral bioavailability of norfloxacin, fluoroquinolone which enter into cells by diffusion [103]. Because of low lipophilicity, diffusion process is very low and the drug is unable to attain therapeutic concentration at the site of infection. Dhaneshwar research group used diglyceride promoiety to enhance bioavailability of poorly absorbed norfloxacin. They synthesized norfloxacin 1,3-dipalmitin ester via coupling of 2-hydroxypropane-1,3-diyl dipalmitate with BOC-protected piperazinyl ring of norfloxacin, followed by deprotection with the TMSBr (Scheme 21). Thus, lipases mediated hydrolysis of the diglyceride ester linkage would release the parent drug norfloxacin in the tissue. The partition coefficient of the novel prodrug **36** determined in chloroform/phosphate buffer reached 5.25 and was 2.7 times higher compared to the parent drug. The release kinetics was examined in vivo in blood, faeces, and urine in Wistar rats’ model. The studies indicated improved pharmacological profile [104].

## Phototherapeutics

Photoresponsive drugs are conjugates of existing biologically active compounds with molecular photoswitches able to undergo remote activation and deactivation. The activity of these drugs can be externally controlled inside the body with light by switching between two or more isomeric states [105]. Local action of such drugs is used to prevent side effects. Velma and coworkers synthesized ciprofloxacin conjugates modified with spiropyran (**37a**) and azobenzene (**37b**) photoswitches by reaction of acyl chlorides of the photoswitches with fluoroquinolone. Spiropyran may be switched to merocyanine form upon 365 nm light irradiation and switched back upon visible-light irradiation or thermal relaxation. The same phenomenon occurs in the structure of azobenzene that undergoes *trans*–*cis* and *cis*–*trans* isomerizations in analogical pattern (Scheme 22). The spiropyran state of the first conjugate **37a** was found to be thermodynamically stable at 555 nm; however, after each round of irradiation significant fatigue was observed, which limits the use of this hybrid to a single round of switching. The other conjugate **37b** exhibited no instability and reversible switching between *cis* and *trans* forms in water. The transformation could be performed more than ten times without any observable fatigue. Evaluation of MIC values in *E. coli* revealed that the spiropyran conjugate **37a** had higher antibacterial activity in its light-induced zwitterionic merocyanine form. This effect may be caused by a change in dipole moment which affects cellular uptake and drug-receptor interactions. In *M. luteus,* no difference before and after irradiation was observed. Azobenzene conjugate **37b** showed the same MIC values in both states tested on *E. coli*, but *trans* isomer had higher anti-microbial activity against *M. luteus* than the *cis* form [106].

**Figure:**
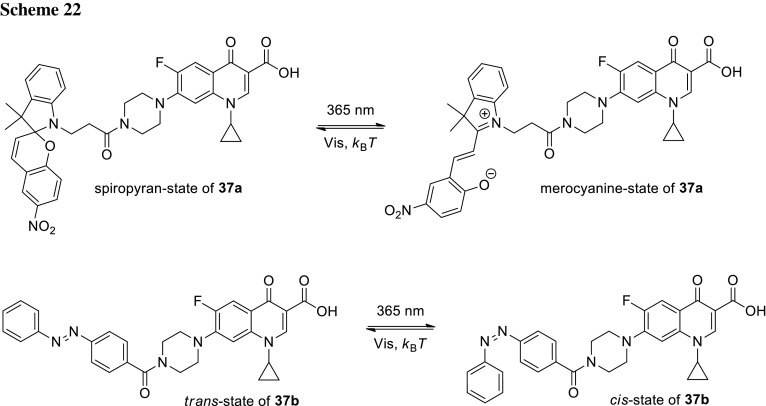

Photodynamic therapy of cancer combines the use of photosensitizing drug, oxygen, and visible light to produce lethal cytotoxic agents like reactive oxygen species (ROS) responsible for the destruction of malignant tissues [107]. Porphyrin derivatives are an example of photosensitizes giving rise to ROS in high yield. Cavaleiro’s research group designed and synthesized porphyrin–quinolone conjugates **38a**–**38d** by 1,3-dipolar cycloaddition of an azidoquinolone to porphyrins bearing alkynyl groups [108]. They also prepared another type of conjugates **39a**–**39d** with use of the Suzuki–Miyaura coupling reaction of a β-borylated porphyrin with bromo-4-quinolones bearing *N*-ethyl and *N*-d-ribofuranosyl substituents and hybrids **40a**, **40b** in the Buchwald–Hartwig reaction between 2-amino-5,10,15,20-tetraphenylporphyrinatonickel(II) and the 6-bromo-4-quinolone substrates followed by an oxidative intracyclization (Scheme 23). The photosensitizing properties of the conjugates **39**, **40** were evaluated in singlet oxygen generation studies. The results were compared to *meso*-tetraphenylporphyrin, well-known singlet oxygen generator. The conjugates **39a**–**39d** and **40c**, **40d** were found to generate singlet oxygen better than the reference photosensitizer [109, 110]. Compounds **40c**, **40d** showed interesting intense absorption bands in the red region of visible spectrum, which makes them potential candidates in PDT. Conjugates **40a**, **40b** were capable of generating singlet oxygen and, however, were slightly less efficient than the standard. Compounds **40a**–**40d** were subjected to photoinactivation tests against *S. aureus* and all of the conjugates were found to be effective anti-microbials. Derivatives **40a** and **40c** were the most active and can be considered to be used in photodynamic inactivation of Gram-positive bacteria [110].

**Figure:**
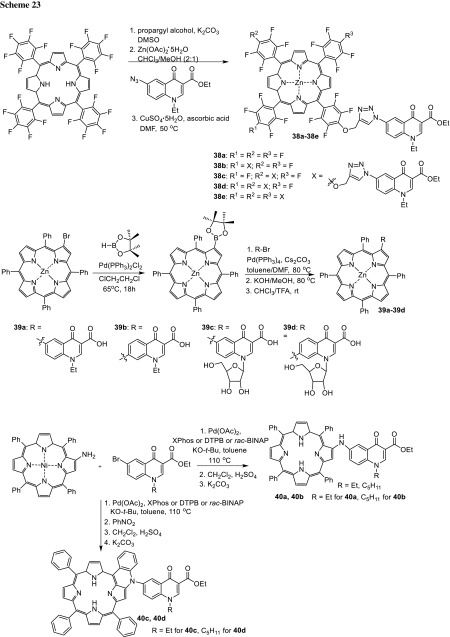

Methylene blue is another example of photoanti-microbial active against a wide range of bacteria, fungi, and viruses [111]. As its structure is related to phenothiazinium, Wainwright and coworkers coupled phenothiazinium derivatives with norfloxacin core to use the obtained conjugates in photoantibacterial targeting. The products were synthesized in reaction between the monosubstituted 3-dialkylaminophenothiazinium intermediates and norfloxacin (Scheme 24). Phenothiazinium compounds **41a**–**41f** absorb light wavelengths in the range of 620–670 nm; thus, singlet oxygen production was measured under red light. In vitro singlet oxygen yields for the hybrids were too low to be determined using the standard spectrophotometric assay employed. The conjugates were found to be much stronger DNA binders and exhibited higher activity against *S. aureus* and *E. coli* bacterial strains than the parent drug after 20 min illumination with 660 nm. Nevertheless, MIC values measured in the foil-covered controls providing dark conditions were high (approximately 100 µM), which suggest lack of essential targeting and does not support the specific DNA-localising hypothesis [112].

**Figure:**
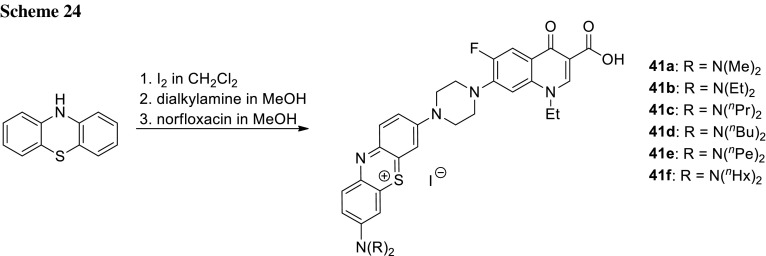

**Fig. 10:**
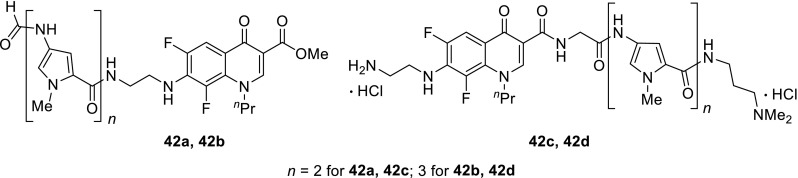
Structures of fluoroquinolone conjugates **41**, **42**

Some of the fluoroquinolones, namely, lomefloxacin and fleroxacin, which have two fluorine atoms, are able to act as photocleavers which upon photoirradiation generate arylcarbene that cause DNA damage. The arylcarbenes exhibit DNA cleaving activity by hydride abstraction from the phosphate backbone of nucleic acid. Suzuki and coworkers combined fluoroquinolone moiety with DNA-binding molecules, di- and tri-(*N*-methylpyrrole) known as DNA minor groove binders. Unexpectedly, the obtained conjugates **42** were photosensitive and undergone gradually decomposition under UV irradiation [113] (Fig. 10).

## Fluorescent compounds

1,8-Naphthylimide derivatives have been described as fluorescent sensors and cellular imaging agents [114]. Kumar and coworkers synthesized hybrids of fluoroquinolone by an aromatic nucleophilic substitution of naphthalimide derivatives with norfloxacin (Scheme 25). Absorption and emission maxima of the conjugates **43** were 338–395 and 505–509 nm, respectively. Fluorescence measured in ethyl acetate, dichloromethane, and chloroform was found to be enhanced in comparison with the free quinolone and red shift of the emission maxima was observed in comparison with 4-bromo-1,8-naphthalic anhydride at excitation wavelength of 380 nm. Antibacterial activity tests were performed against *E. coli* and *S. aureus* strains and compound **43b** showed the highest inhibition of both bacterial strains tested. Docking studies with ATP-binding pocket of *E. coli* topoisomerases (Gyrae B and ParE) revealed that this compound exhibits the highest binding affinity to ATP-site. The results described above make the conjugates potential drug candidates [115].

**Figure:**
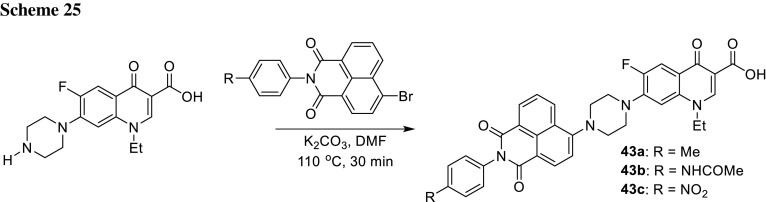

## Triazoles

Substituted triazoles demonstrate considerable activity towards Gram-positive and Gram-negative bacteria [116] and are very well-recognized pharmacophores [117]. Ozdemir and coworkers performed Mannich condensation of 1,2,4-triazole-3-thioles with a secondary amine groups of piperazinyl moiety within norfloxacin (**44a**–**44c**) or ciprofloxacin (**44d**–**44f**) (Scheme 26). Anti-microbial activity of the conjugates was evaluated against *E. coli*, *Yersinia pestis*, *P. aeruginosa*, *S. aureus*, *E. faecalis*, *B. cereus*, and *M. smegmatis*. The conjugates exhibited excellent activity towards tested strains with MIC values between 0.24 and 1.9 μg/cm^3^, comparable to the parent quinolones [118].

**Figure:**
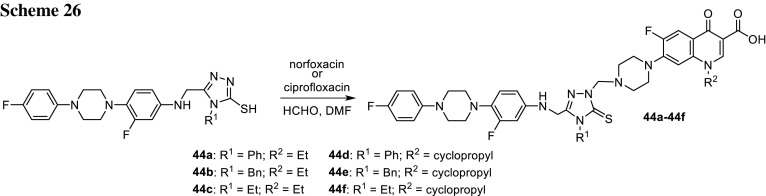

**Fig. 11:**
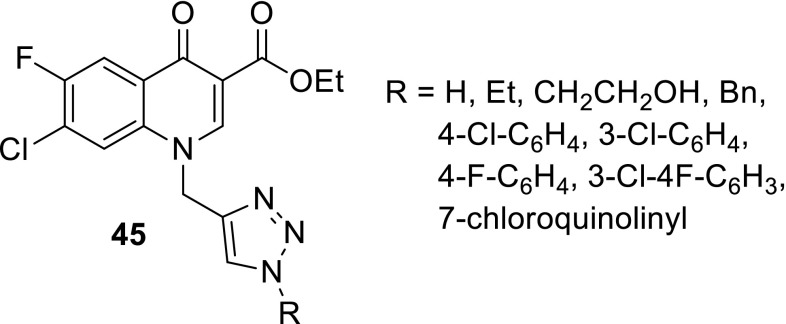
Structures of triazole–fluoroquinolone hybrids **45**

Dixit and coworkers employed click chemistry to synthesize fluoroquinolone analogs **45** bearing triazole ring on N1 nitrogen atom. The obtained compounds were tested against malaria parasite, *P. falciparum*. The compound **45** with unsubstituted triazole ring (R = H) was found to be the most active with IC_50_ of 1.33 µg/cm^3^, and proved to be almost sevenfold more potent than ciprofloxacin. In vitro cytotoxicity experiments revealed that this compound was the least toxic among screened compounds against HEK-293 cells [119] (Fig. 11).

Fluoroquinolones bearing triazole moiety linked to piperazinyl ring **46**–**48** were also analyzed. Ciprofloxacin (R^1^ = cyclopropyl; R^2^ = F; X = CH; Y = C), norfloxacin (R^1^ = Et; R^2^ = F; X = CH; Y = C), and pipemidic acid (R^1^ = Et; X = Y = N) were converted to alkyne derivatives, then subjected click reactions with amino-acid azides (**46a**–**46g**), dipeptide azide (**47a**–**47c**), or aryl azides (**48a**–**48i**) utilizing the microwave-assisted technique (Scheme 27). The conjugates were obtained in fair yields (40–72%) and tested against *S. aureus*, *Staphylococcus pyogenes*, *Salmonella typhi*, *P aeruginosa*, and *E. coli*. The compounds possessing aryl substituents **48a**–**48i** were the most active within the series [120].

**Figure:**
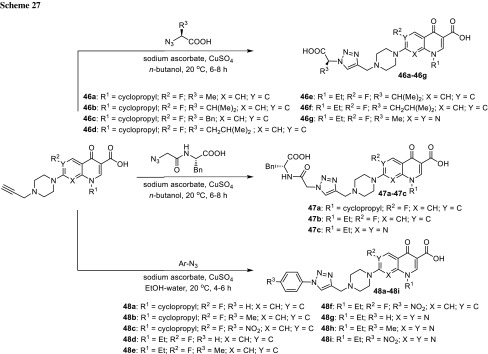

Plech and coworkers also synthesized ciprofloxacin derivatives **49** with triazole bind to the piperazinyl ring. They obtained 40 novel conjugates through Mannich reactions in yields of 63–80% (Scheme 28). The compounds showed an increase of lipophilic properties (logP of obtained conjugates of 1.63–4.62 vs. − 0.70 determined for ciprofloxacin). Most of the derivatives were found to be more active than the parent ciprofloxacin against strains causing life-threatening infections (i.e., *S. aureus*, *S. epidermidis*, *B. subtilis*, *B. cereus*, *M. luteus*, *E. coli*, *Proteus mirabilis*, and *P. aeruginosa*), despite the fact that the majority of the conjugates were found to be weaker DNA gyrase and topoisomerase IV inhibitors than the parent drug in enzymatic studies. This high antibacterial activity may be caused by easier permeation inside bacterial cells or by the fact that these agents are not substrates or are poorer substrates for the bacterial endogenous efflux systems. Cytotoxicity of the selected compounds was determined towards HEK-293 cells using MTT assay and was found to be remarkably lower than MIC values [121–123].

**Figure:**
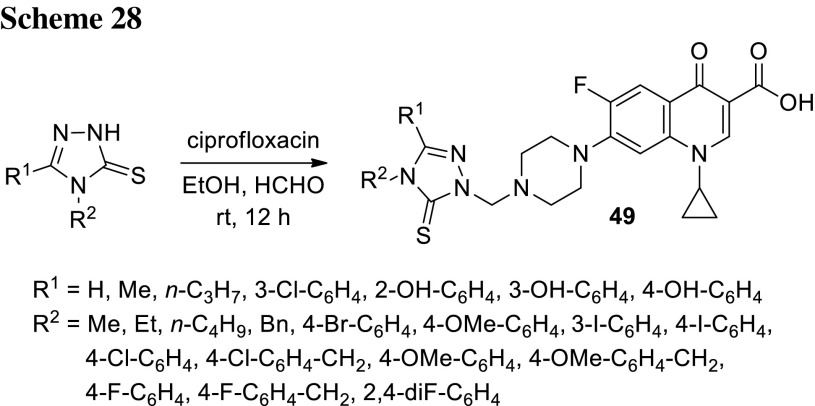

Kant and coworkers synthesized bis-triazole conjugates of ciprofloxacin **50a**–**50r** in two-step procedure. The first step involved the reaction of ciprofloxacin with propargyl bromide in the presence of NaHCO_3_ at 100 °C. In the next step, the obtained intermediate was reacted with substituted aromatic azides via copper-catalyzed azide-alkyne cycloaddition that afforded the desired products (Scheme 29). All hybrids were screened in vitro for their antibacterial activity against *S. aureus*, *S. epidermidis*, *E. faecalis*, *E. coli*, *P. aeruginosa*, *Aeromonas hydrophila*, *S. typhimurium*, *S. typhi*, *Sphingomonas paucimobilis*, and *Plesiomonas shigelloides.* Compared to the parent drug, compounds **50h**, **50j**, **50m** displayed two to tenfold more potent activity against the tested species. The structure activity relationship revealed that the novel compounds with strong electron withdrawing substituents at the position 4 of the benzene ring enhanced the anti-microbial action, while electron-releasing groups decreased inhibitory activity. Hemotoxicity studies of the evaluated compounds revealed negligible toxicity profiles [124].

**Figure:**
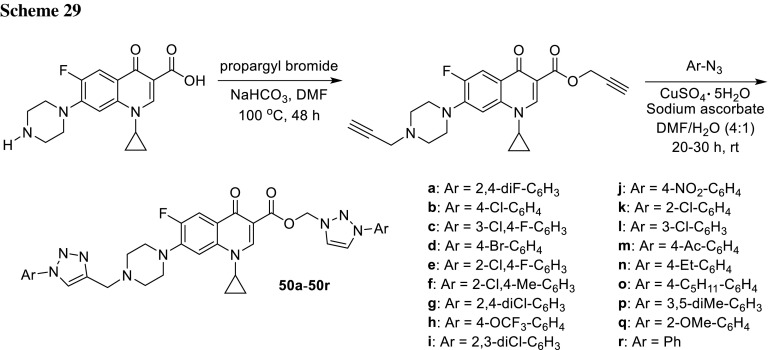

**Fig. 12:**
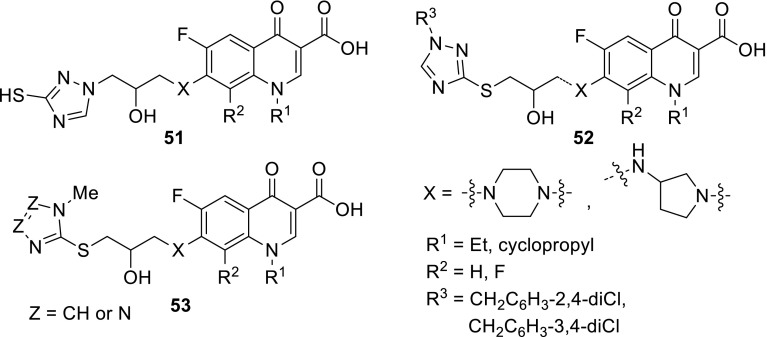
Structures of fluoroquinolone–triazole hybrids **51**–**53**

Zhang and coworkers prepared a series of novel azolylthioether modified quinolones **51**–**53**. Azolylthioethers constitute a class of structural fragments common in many natural and synthetic active compounds exerting their function by transforming thioether into thiol moiety that disrupt structural integrity of many important proteins and deregulate the rate of intracellular oxidoreductive species [125]. In addition, the sulfur moiety is able to improve lipophilicity and moderate electron density of azole ring as an electron-rich center, thereby influencing the transmembrane diffusion ability. Commercially available quinolones were subjected reaction with 2-(chloromethyl)oxirane and subsequently reacted with azoles to produce the target compounds **51**–**53** in 21–35% yield. Anti-microbial potency was tested in vitro against a panel of bacteria and fungi (*Cyberlindnera utilis*, *Aspergillus flavus*, *Saccharomyces cerevisiae*, *Candida albicans*, *Candida mycoderma*, *M. luteus*, *S. aureus*, *B. subtilis*, *P. aeruginosa*, *E. coli*, *P. vulgaris*, and *Escherichia typhosa*). Biological activities of the obtained compounds were high, especially for derivative **53** (Z = CH, X = pyrrolidin-3-amine, R^1^ = cyclopropyl, R^2^ = F) with the lowest MIC values of 0.25 µg/cm^3^ against *S. aureus* MRSA and *P. aeruginosa*, superior to reference drugs. Docking studies with topoisomerase IV and genomic DNA form *S. aureus* MRSA revealed that this compound could interfere with nucleic acid through a copper-ion bridge to form a steady ternary complex which might block DNA replication. Moreover, studies of resistance development for compound **52** (X = piperazine, R^1^ = Et, R^2^ = H, R^3^ = CH_2_C_6_H_3_-2,4-diCl) revealed that after 25 passages bacterial resistance towards *S. aureus* MRSA did not increase, while norfloxacin showed increase of resistance after six passages [126] (Fig. 12).

## Hybrid drugs

**Fig. 13:**
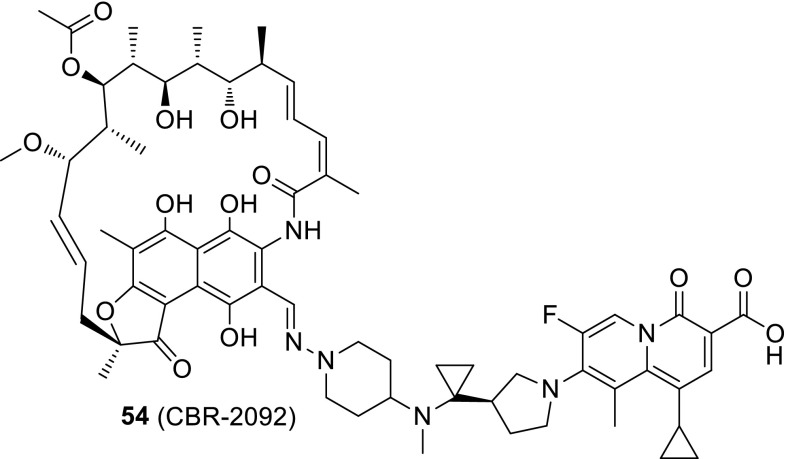
Structure of rifamycin–quinolone conjugate **54** (CBR-2029)

Combining of two existing drugs into one molecule is a common approach to extend biological activity and avoid antibiotic multidrug resistance caused by microorganism mutations. CBR-2029 (**54**) is a novel hybrid antibiotic with a structure of covalently combined rifampicin and quinolone. Rifamycin drugs inhibit bacterial DNA-dependent RNA synthesis, exhibit excellent tissue distribution [127], and efficiently penetrate biofilm formed in vitro [128]. Recent studies demonstrated that CBR-2029 exhibit prolonged bactericidal activity against *S. aureus*, superior to rifampicin, ciprofloxacin, moxifloxacin, or cocktail of rifampicin and moxifloxacin. Moreover, this drug did not cause resistance development and was not a substrate for the NorA or MepA efflux pumps of *S. aureus* [129]. The CBR-2029 potency of *S. aureus* RNA polymerase inhibition was twofold less than rifampin (IC_50_ of 0.034 µM vs. 0.015 µM for the reference drug); however, this drug exhibited nearly equipotent activity against *S. aureus* DNA topoisomerase IV and gyrase as ciprofloxacin and gatifloxacin (IC_50_ values of 1.7 and 1.5 µM for corresponding enzymes, respectively). CBR-2092 revealed delayed additional mode of action: i.e., rifampin-like effect on protein and cell wall synthesis [130] (Fig. 13).

**Fig. 14:**
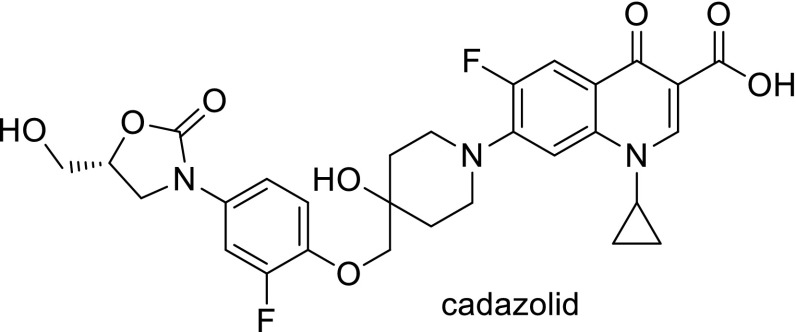
Structure of cadazolid

Oxazolidones are class of antibiotics that act at early step in bacterial protein synthesis. Cadazolid (Fig. 14) is an experimental drug bearing both oxazolidinone and fluoroquinolone moiety, invented by Actelion Pharmaceuticals Ltd. Recently, it had been extensively investigated for the treatment of *Clostridium difficile*, a Gram-positive toxin- and spore-forming anaerobe which is the most common cause of antibiotic-associated diarrhea and colitis. Cadazolid was tested in vitro against references as well as clinical isolates of *C. difficile* with an MIC range of 0.125–0.5 µg/cm^3^ [131]. The propensity of spontaneous resistance development found to be low; MIC values in tested strains increased only very slowly upon 13 passages [132]. Inhibition of translation was tested in biochemical assays and it was determined as the primary mode of action of cadazolid in *C. difficile*. Cadazolid potently inhibited protein synthesis in wild type and quinolone-resistant strains. Inhibition of DNA synthesis was suggested as a second mode of action, which was proved on the basis of macromolecular labeling studies. Inhibitory effects in DNA topoisomerase assays were weak. Concentrations needed to observe that half-maximal inhibition of DNA synthesis was more than 60-fold higher than these for protein synthesis inhibition [132]. Cadazolid strongly inhibited toxin formation and delayed the formation of spores at sub-growth-inhibitory concentrations. Tests in mouse and hamster models for prevention of diarrhea and mortality showed that cadazolid markedly decreased the risk of death [131]. Cadazolid demonstrated narrow spectrum activity eliminating *C. difficile* while having a very limited impact on the normal gut microbiota. In the in vitro gut model, it rapidly reduced counts of vegetative cell and cytotoxin titers of *C. difficile* in simulated infection, which declined to the limit of detection by the end of the 7-day dosing period, and showed no sign of recurrence. Regimen of 250 mg/dm^3^ did not appear to have a more inhibitory effect on microflora populations. Cadazolid application provoked sparing of the enumerated gut microflora with the exception of bifidobacteria and only a slight decreases in total *Clostridium* and *Enterococcus* populations [133]. Phase 1 clinical trials were performed in total of 64 healthy male subjects in single or multiple (twice daily for 10 days) oral doses of cadazolid between 30 and 3000 mg, or placebo. After first trials in humans cadazolid found to be well tolerated and its systemic exposure was low. The high concentrations of the drug were found at the site of action (colon). Probably due to its poor water solubility, cadazolid mostly retained in the gastrointestinal tract after oral administration. The lack of metabolites or potential degradation products was detected in plasma and majority of the compound was recovered unchanged in the faeces within 72 h of single oral administration, which together suggests high stability of the compound in the tested human matrices [134]. In the phase 2 clinical study, efficacy and safety of three oral dosages of cadazolid (250, 500, or 1000 mg) were investigated in comparison with vancomycin (reference drug) in 84 patients with *C. difficile* infections. The cure rates for both drugs were similar: 68.2% for vancomycin and 68.4–80.0% for cadazolid. The recurrence rates were lower with all cadazolid dosages (18.2–25.0%) than with vancomycin (50.0%) [135]. Moreover, the susceptibilities of *C. difficile* isolates to cadazolid were evaluated. The MIC values of epidemic strains isolated from patients from phase 2 clinical trial were low an in the narrow range. Even for the lowest dosage of cadazolid, the faecal concentration of the drug was in higher than MIC for *C. difficile* [136]. To our best knowledge, results from phase 3 trials have not been published yet and are still analyzed.

Darekhordi and coworkers synthesized oxazolidone derivatives of fluoroquinolones **55** via amination of *N*-aryltrifluoroacetimidolyl chlorides with norfloxacin or ciprofloxacin piperazinyl group (Scheme 30). The conjugates were obtained in good yields (60–86%) and some of them were selected to antibacterial activity tests performed against *S. aureus*, *E. coli,* and *K. pneumoniae*. Compounds **55a** and **55c** were found to be better antibacterial agents than ciprofloxacin against all strains tested at concentrations of 10–15 µg/cm^3^ in agar diffusion test [137].

**Figure:**
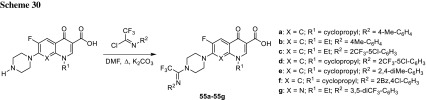

Azithromycin is one of the macrolide antibiotics that act by inhibition of bacterial protein synthesis. A series of azithromycin–quinolone hybrids **56a**–**56f** linked with ether linkers were synthesized in the route, as presented in Scheme 31. Antibacterial activities of the hybrids were tested on *S. aureus*, *Streptococcus pneumoniae*, and *S. pyogenes.* Most of the conjugates were poorly active; however, compound **56c** exhibited the highest antibacterial activity against tested strains [138].

**Figure:**
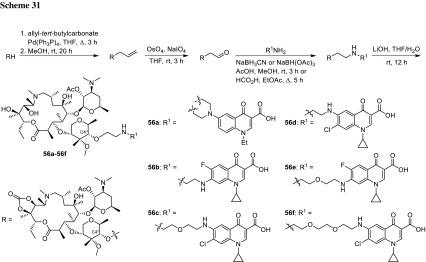

**Fig. 15:**
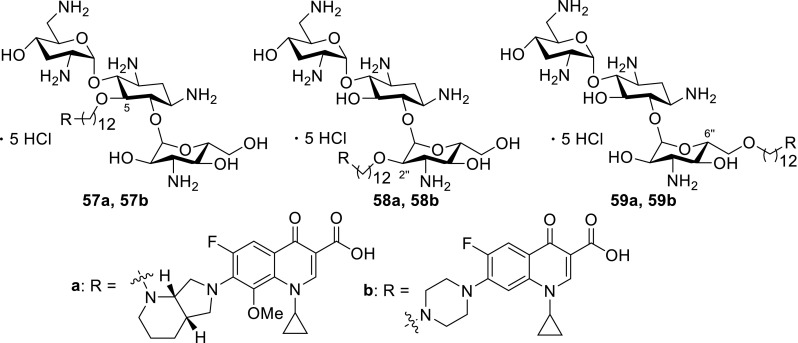
Structures of tobramycin–fluoroquinolone conjugates **57**–**59**

Another antibiotic used in fluoroquinolone conjugates is tobramycin. This drug belongs to broad-spectrum antibiotic aminoglycosides that works by binding to a site on the bacterial 30S and 50S ribosome units preventing the formation of the 70S complex and can cause outer membrane disruption [139]. Gorityala and coworkers synthesized conjugates **57**–**59** of ciprofloxacin and moxifloxacin with tobramycin linked by long carbon chains and evaluated antibacterial properties of the obtained conjugates. Hybrids **58a**, **58b** and **59a**, **59b** showed weak antibacterial action; nevertheless, compounds **57a**, **57b** were found to be good antibacterials, even against resistant *P. aeruginosa* strains. The most active conjugates **57a**, **57b** demonstrated an ability to destabilize membrane and better inhibit DNA gyrase A and topoisomerase IV than the parent fluoroquinolone. However, reduction in protein translation inhibition was observed. The hybrids displayed also delayed bacterial resistance development, low cytotoxicity against cancer cell lines, and hemolysis of human erythrocytes below 10% [140, 141]. Moxifloxacin derivative **57a** showed no toxic effect in *Galleria mellonella* up to the maximal dose of 600 mg/kg. Efficacy studies in larvae infected with XDR *P. aeruginosa* strain revealed 100% survival after 24 h with single-dose therapy of 50 mg/kg and enhanced the long-survival effect, while treatment with moxifloxacin or tobramycin resulted in 20–27% survival [141] (Fig. 15).

**Fig. 16:**
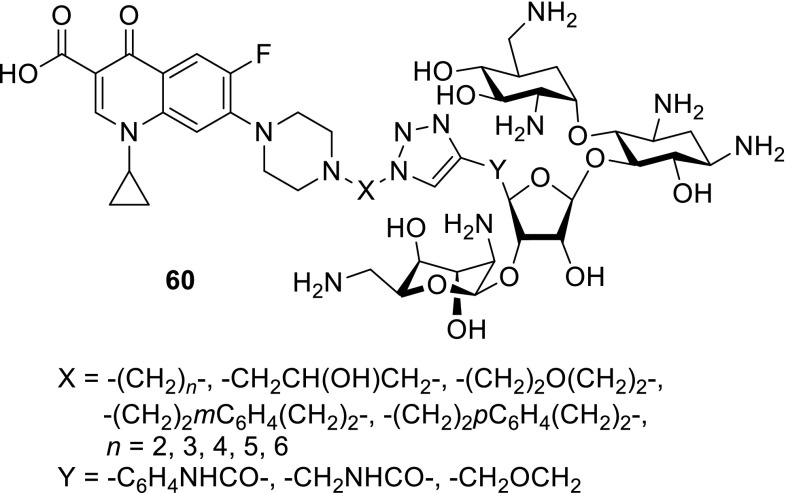
Structures of ciprofloxacin–neomycin B hybrids **60**

Pokrovskaya and coworkers also used aminoglycosides to form dual antibiotics with fluoroquinolones. They coupled ciprofloxacin–azide and neomycin B-alkyne derivatives via *click* chemistry to afford a library of 17 conjugates **60** with different spacer lengths. The reactions were performed under microwave irradiation in the presence of an organic base and the Cu(I) catalyst in excellent yields. Antibacterial activity of the obtained compounds in MIC assays was improved in comparison with neomycin B, however, lower than ciprofloxacin. Inhibition of DNA gyrase, topoisomerase IV, and protein synthesis of the most potent hybrids revealed that the novel compounds are better enzyme inhibitors than both parent drugs confirming desired dual mode of action. Higher MIC values might be a consequence of reduced cell penetration by the conjugates with higher molecular weights [142] (Fig. 16).

Quinine is a unique therapeutic agent with exceptional pharmacological efficacy as an antimalarial drug. Panda and coworkers prepared group of conjugates **61**, **62** that comprised quinolone antibiotics, quinine and amino-acid linkers utilizing benzotriazole chemistry (Scheme 32). They used levofloxacin, enrofloxacin, oxolinic acid, and nalidixic acid as precursors of the conjugates to enhance the antimalarial activity of the drugs. The obtained hybrids retained in vitro antimalarial activity with IC_50_ values ranging from 12 to 207 µM determined in antimalarial bioassay against *P. falciparum* 3D7, chloroquine-sensitive strain. The results were comparable to that assessed for quinine (IC_50_ = 18 µM) [143].

**Figure:**
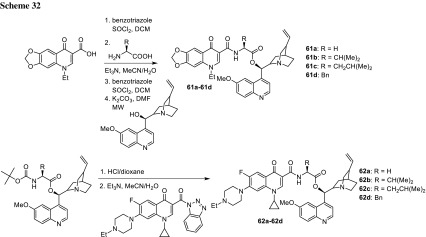

The same research group in a similar way prepared fluoroquinolone–pyrazine hybrids **63a**–**63h** with amino-acid linkers. Pyrazinamide is one of the first-line antituberculosis drugs which has multiple mechanisms of action. It acts as a prodrug, since it is metabolized to pyrazinoic acid via mycobacterial enzyme pyrazinamidase [144]. Linkers between biologically active compounds were used to modify lipophilicity and increase drugs ability to penetrate into mammalian tissue. The novel compounds were synthesized in acetonitrile under microwave irradiation at 20 W, 50 °C for 1 h, by coupling amino acid or γ-aminobutyric acid–fluoroquinolone conjugates of norfloxacin or ciprofloxacin with (1*H*-benzo[*d*] [1,2,3] triazol-1-yl)(pyrazin-2-yl)methanone in the presence of DBU (Scheme 33). Subsequently, they were investigated against *S. typhi*, *P. aeruginosa*, *S. aureus*, and *S. pyogenes* bacteria. The ciprofloxacin derivative **63h** was found to be the most promising antibacterial agent against *S. aureus* ATCC29523 and *S. pyogenes* ATCC19615 with MIC values 74.6 and 149.3 µM, respectively. These two strains were resistant to both reference drugs. However, **63h** was less active than investigated fluoroquinolones against *S. typhi* and *P. aeruginosa* bacteria, which were susceptible to both norfloxacin and ciprofloxacin [145].

**Figure:**
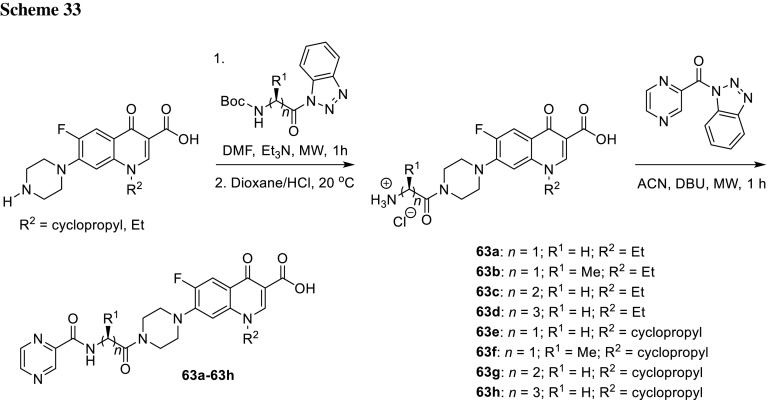

**Fig. 17:**
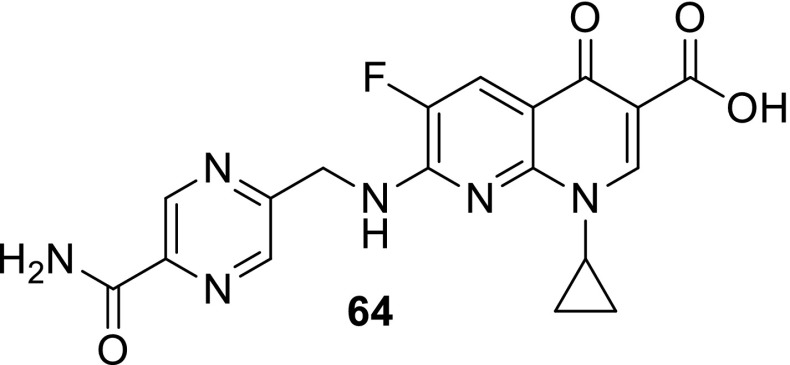
Structure of quinolone–pyrazinamide hybrid **64**

Markad and corworkers investigated quinolone–pyrazinamide hybrid **64** as potential antituberculosis drug candidate. This compound exhibited improved antitubercular activity in comparison with pyrazinamide and wide antibacterial action against common bacterial pathogens; however, it was inactive in DNA supercoiling assay indicating novel mechanism of action [146] (Fig. 17).

**Fig. 18:**
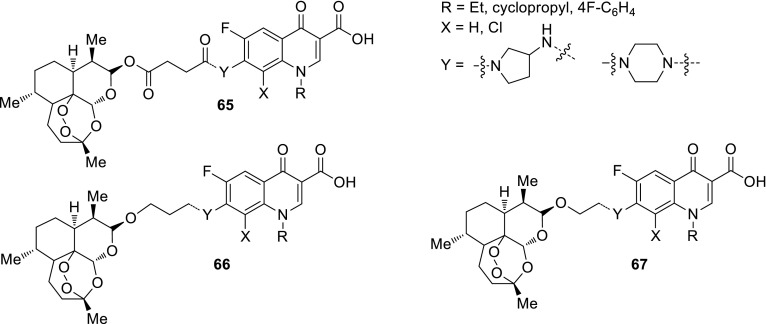
Structures of dihydroartemisinin–fluoroquinolone conjugates **65**–**67**

Zhou and coworkers used dihydroartemisinin (DHA) to produce fluoroquinolone conjugates **65**–**67** aiming to treat tuberculosis. DHA is artemisinin derivative exhibiting better solubility, bioavailability, and biological activity than artemisinin. Both drugs find applications as antimalarial agents [147]; however, DHA was under investigation for its antitubercular activity and showed potent inhibition of *Mycobacteria.* Ciprofloxacin, norfloxacin, sarafloxacin, and clinafloxacin were coupled with DHA derivatives and subjected anti-microbial experiments. The majority of the newly synthesized conjugates **65**–**67** were active and selective against *Mycobacterium tuberculosis*. Clinafloxacin derivatives **66** and **67** (R = cyclopropyl, X = Cl, Y = pyrrolidin-3-amine) exhibited stronger activity than the parent drug and were extremely potent against reference strain as well as clinical isolates, both sensitive and multidrug resistant [148] (Fig. 18).

Sriram and coworkers designed tetracycline–fluoroquinolone hybrids which were investigated for their antimycobacterial and antiviral properties. As tetracycline exhibit HIV-1 integrase inhibitory activity, they hypothesized these hybrid compounds could act both as HIV-1 integrase inhibitors and as antibiotics in *M. tuberculosis* treatment. Compounds **68a**–**68d**, **68j** were found to be excellent anti-HIV agents preventing virus replication and exhibited lower cytotoxicity against CEM cell line, while conjugates **68d**, **68j**, and **68l** exhibited high activity against *M. tuberculosis* [149] (Scheme 34).

**Figure:**
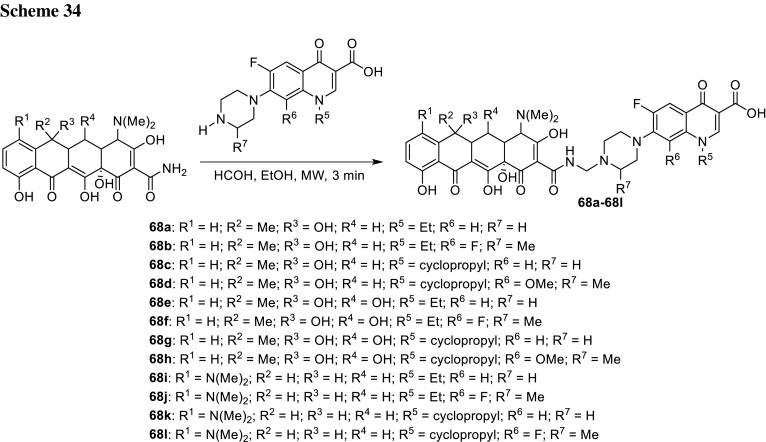

Metronidazole is an example of synthetic antibiotic. This drug demonstrates inhibitory efficacies against Gram-negative anaerobic bacteria, such as *Helicobacter pylori*, and protozoa, i.e., *Giardia*, *Lamblia*, and *Entamoeba histolytica* [150]. Nitro fragment present in its structure undergoes reduction process by metabolic pathway and the reactive intermediate is able to damage nucleic acids [151]. The conjugates of metronidazole and quinolones **69**, **70** were prepared by Cui and coworkers in multi-step synthesis (Scheme 35) and evaluated in vitro against Gram-positive (*S. pneumoniae*, *M. luteus*, 2 strains of *S. aureus*, and *B. subtilis*) and Gram-negative (*P. aeruginosa*, *E. coli*, *Shigella dysenteriae*, and *E. typhosa*) bacteria as well as fungi (*C. albicans*, *C. mycoderma*, and *A. flavus*). Antifungal activity of the obtained compounds was found to be moderate or weak. The measured MIC values were in the range of 64–512 µg/cm^3^ only with small exception for the hybrids **69b** and **70b**, which were more potent than the reference drug, fluconazole. The synthesized compounds exhibited divergent activity in antibacterial assays. The most potent conjugates were compounds **70b** and **70d** (MIC values of 0.5–8 µg/cm^3^). Hybrids **69b**, **70b**, and **70d** were further examined for their cytotoxic properties on A549 and normal human hepatocyte LO2 cell lines and no obviously reducing trends to the cell viability were observed within the concentration of 128–256 µg/cm^3^. Molecular modeling studies were performed with use of the crystal structure of *S. pneumoniae* topoisomerase IV–DNA complex and the docking results of the target hybrids showed that the substituents on benzene ring of quinolone could affect the antibacterial activities. Compound **70d** was tested in binding assay with calf-thymus DNA and exhibited stronger interferation with nucleic acid than norfloxacin. These conjugates exhibited also good aqueous solubility, which combined make them good potential drug candidates [152].

**Figure:**
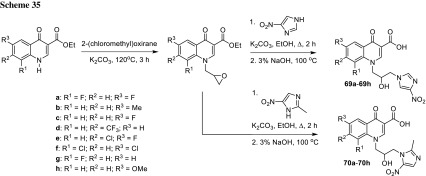

**Fig. 19:**
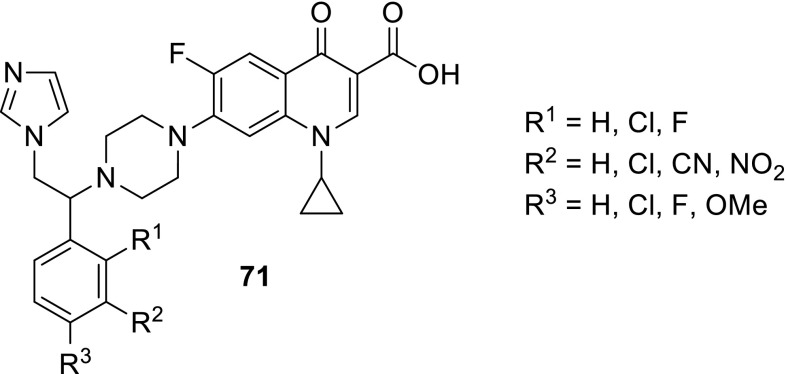
Structures of miconazole-based ciprofloxacin conjugates **71**

Miconazole is a well-established drug that acts by competitive inhibition of the cytochrome P-450 enzyme through direct intercalation [153]. This action leads to lethal disruption in the normal sterol biosynthesis chain in fungi. Gu and coworkers designed miconazole-based ciprofloxacin conjugates **71**. Antibacterial and antifungal activities of the newly prepared compounds were evaluated in vitro against Gram-positive (*M. luteus*, *B. subtilis*, and 2 strains *S. aureus*), Gram-negative (*P. aeruginosa*, *E. coli*, and *P. vulgaris*) as well as fungi (*C. albicans*, *C. mycoderma*, *C. utilis*, and *S. cerevisiae*). Compound **71** (R^1^ = F, R^2^ = H, R^3^ = F) was found to be comparable or more potent than ciprofloxacin and miconazole in antibacterial and antifungal assays, respectively [154] (Fig. 19).

Fluconazole belongs to triazole antibiotics recommended by WHO as the first-line antifungal drug. Wang and coworkers prepared conjugates of clinafloxacin with fluconazole-like substituents **72** in a reaction of fluoroquinolone with the corresponding oxiranes (Scheme 36). The obtained hybrids were tested against a panel of Gram-positive and Gram-negative bacteria as well as fungi. In general, the novel conjugates exhibited good anti-microbial efficacies with MIC values of 0.5–32 µg/cm^3^. The modification not only effectively increased their biological activities and broadened their spectrum of action in comparison with the precursor clinafloxacin and fluconazole, but also improved their physiochemical properties and water solubility [155].

**Figure:**
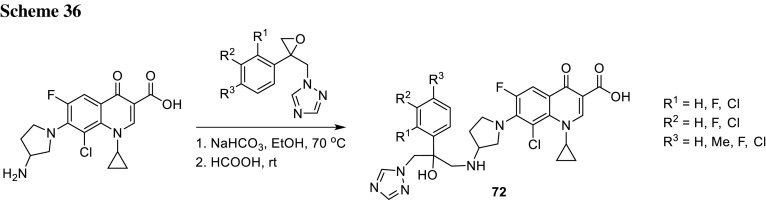

## Bis-quinolones

Fluoroquinolones were also used as homodimeric and heterodimeric antibiotic agents. Panda and coworkers synthesized novel quinolone–fluoroquinolone conjugates with amino-acid linkers **73**–**76** by means of benzotriazole chemistry in good yields (60–82%) (Scheme 37). The obtained conjugates exhibited weak antibacterial activity against *S. aureus*, *S. pyogenes*, *S. typhi*, and *P. aeruginosa*, in most cases comparable with the parent drugs. However, the highly active compounds **73b** and **73f** were found to be much more potent than the parent fluoroquinolones against *S. pyogenes* and *S. aureus*, respectively [156].

**Figure:**
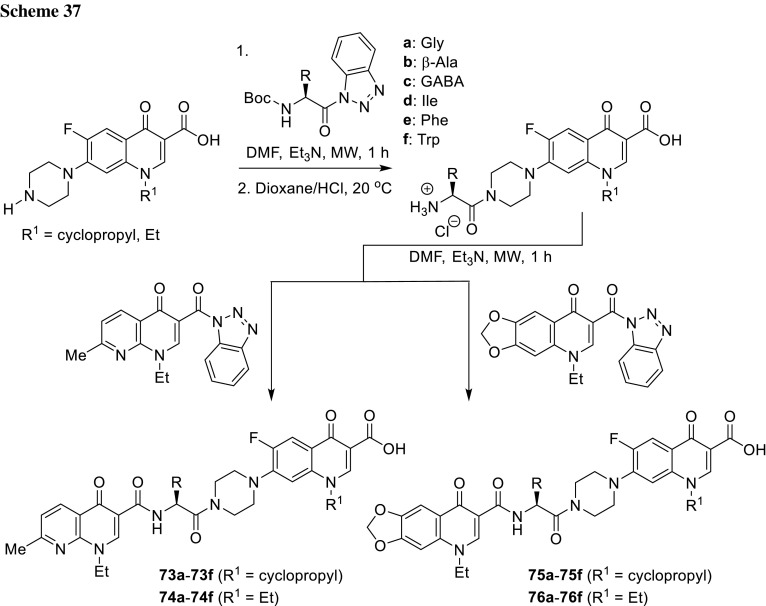

**Fig. 20:**
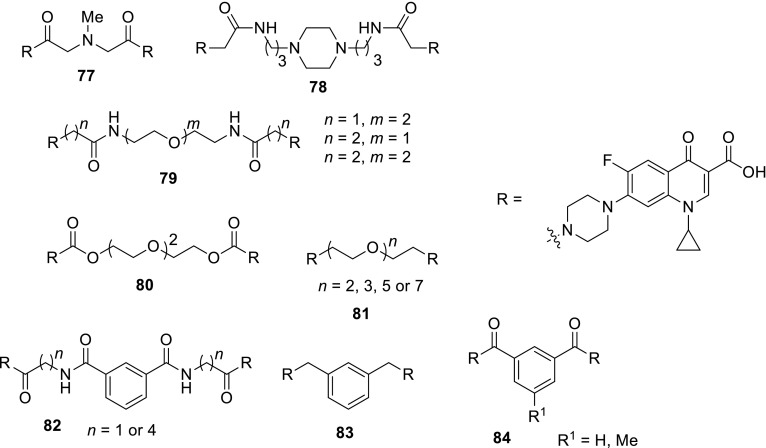
Structures of ciprofloxacin dimers **77**–**84**

Ross and coworkers designed amine- (**77**, **78**), peptoidal- (**79**), PEG- (**80**, **81**), or aryl-linked (**82**–**84**) ciprofloxacin dimers. The dimers were tested for biochemical inhibition of *E. coli* DNA gyrase; however, only for the compound **84** (R^1^ = H) the inhibition activity was not suppressed. All the conjugates exhibited MIC values above or comparable to the parent monomer in assays performed against *E. coli*, *P. aeruginosa*, and *S. aureus*. However, efflux-deficient mutant JW5503-1 was substantially more susceptible to all compounds, which indicate that the dimers remained efflux pump substrates [157] (Fig. 20).

Azema and coworkers designed C7/C7-linked ciprofloxacin (**85**, **86**) and C6/C6-linked levofloxacin (**87**) dimers. The conjugates of ciprofloxacin were synthesized via direct acylation of fluoroquinolone drug with carboxylic di-acids activated by EDCl and 1-hydroxybenzotriazole in DMF/dichloromethane mixture or in condensation of carboxylic di-acids with ciprofloxacin derivative in yields ranging from 31 to 70% (Scheme 38). Levofloxacin hybrids were obtained in the procedure involving an in situ-generated acyl chlorides, which were subsequently reacted with diamines. All the conjugates were evaluated for their in vitro inhibition of human cancer cells (pro-apoptotic stimuli U373-MG glioblastoma and A549 NSCLC as well as apoptosis-sensitive the PC-3 prostate, the LoVo colon and the MCF-7 breast carcinoma cell lines) in MTT assays. Compounds **85** (*n* = 14), **86** (*n* = 14), and **87** (*n* = 9) were found to be the most potent (IC_50_ values below 10 µM). The anti-proliferative activity increased with the alkyl linker length; however, loss of activity was observed in compounds exhibiting poor solubility (conjugates with long linker chains tended to precipitate). Anti-microbial activity of the hybrids was tested against *S. aureus*, *Enterococcus hirae*, *E. coli*, *P. aeruginosa*, and *M. tuberculosis.* The conjugates of ciprofloxacin showed moderate-to-weak activity against bacterial and mycobacterial strains, lower than that found for the parent monomer. Only compound **87** (*n *= 4), proved to be more active against *S. aureus* strains than ciprofloxacin [158].

**Figure:**
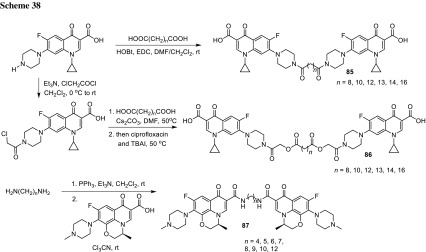

C3/C3 heteroconjugates of fluoroquinolones cross-linked with [1,2,4] triazolo[3,4-*b*] [1,3,4] thiadiazole **88**, **89** were prepared by Hu and coworkers. The conjugates were synthesized with use of ciprofloxacin or its *N*-methyl and *N*-ethyl derivatives obtained through cyclocondensation of fluoroquinolone drug with carbon disulfide followed by base-catalyzed conversion into the 4-amino-4*H*-1,2,4-triazole-3-thiols (Scheme 39). Coupling of the intermediate with other fluoroquinolones (ciprofloxacin, enrofloxacin, norfloxacin, ofloxacin, and levofloxacin) was performed with use of POCl_3_ (Scheme 39). The bis-fluoroquinolones were subjected to antitumor activity tests against murine leukemia (L1210), human leukocytoma (HL60) and Chinese hamster ovary (CHO) cell lines with the use of the MTT assay. The determined IC_50_ values were in the range 0.12–26.2 µM. The ciprofloxacin–ciprofloxacin conjugate **88** (R^1^ = R^3^ = H, R^2^ = cyclopropyl) and ciprofloxacin–levofloxacin **89** (*S*-(–)-R^1^ = H) showed the highest antitumor activity against HL60 cell line (IC_50_ values of 0.54 and 0.12, respectively) [159].

**Figure:**
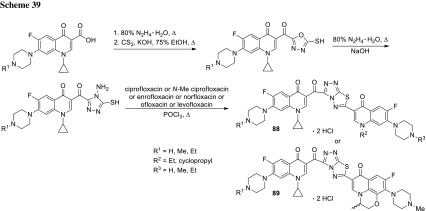

## Other modifications

Conjugation of fluoroquinolone with isatin had been proposed to improve the drug lipophilicity in treatment of *Mycobacteria* [160–164]. 8-Methoxyciprofloxacin was modified with methylene and ethylene derivatives of isatin bearing oxime, methyloxime, ethyloxime, semicarbazone, and thiosemicarbazone moieties (Scheme 40). The methylene conjugates **90** were obtained by Mannich reactions of substituted isatine, paraformaldehyde and 8-methoxy ciprofloxacin in refluxing alcohol under an argon atmosphere, while ethylene compounds **91a** were synthesized via nucleophilic substitutions in DMF at 40 °C and subsequent condensations with the corresponding amine hydrochlorides to form Schiff’s bases **91b**. The conjugates were obtained in moderate-to-high yields (40–85%). The calculated log*P* values were assessed in the range of 1.26–3.33 vs. 1.20 for the parent drug, which indicated remarkable improvement in the drug lipophilicity. This trend was reproduced for the experimental log*P* values determined by HPLC technique: 1.08–1.67 for the conjugates and 0.79 for 8-methoxyciprofloxacin. The conjugates were tested against *M. smegmatis* CMCC 93202 using serial double dilution technique in duplicate as well as against *M. tuberculosis* H37Rv ATCC 27294 and multidrug-resistant clinical isolate *M. tuberculosis* 09710 using a rapid direct susceptibility test technique. Most of the compounds exhibited relatively good activity against *M. smegmatis* strain, but they were less active than the parent quinolone. Nevertheless, in *M. tuberculosis* assays the conjugates were found to be notably more potent than the reference drug. Compound **90** (R = F, R^2^ = O) was found to be highly active against reference *M. tuberculosis* (MIC: 0.074 µM), while conjugates **90** (R = F, R^2^ = NOMe; R = H, R^2^ = NNHCONH_2_; R = F, R^2^ = NNHCONH_2_; R = H, R^2^ = NNHCSNH_2_) were extremely potent against the multidrug-resistant strain (MIC 6.72–7.05 µM). Toxicity was examined in mammalian Vero cell line and the selectivity index for the most active compounds was assessed in the range of 954–1902, which make these compounds attractive potential therapeutic agents [160]. 1,2,3-Triazole-tethered ciprofloxacin–isatin hybrids **92a** (R^1^ = R^2^ = H) were also designed and synthesized by Cu-promoted azide-alkyne cycloaddition in the presence of CuI and trimethylamine in acetonitrile/dichloromethane mixture giving rise to the formation of the desired products **92a** in 22–35% yield. The resulted products were transformed into metoxime derivatives **93** via condensation with methoxyamine hydrochloride in the presence of NaHCO_3_ (32–44% yield). All hybrids were less active than ciprofloxacin active against *M. smegmatis* (MIC values of conjugates in the range of 12.5–100 vs. 6.25 µg/cm^3^ for ciprofloxacin) and exhibited comparable activity to the parent drug against *M. tuberculosis* H37Rv (1.56–25  vs. 3.12 μg/cm^3^). Fluorine metoxime derivative **93** (R = F, R^1^ = R^2^ = H; R^3^ = OMe) was twice more potent than the parent drug against *M. tuberculosis* (MIC 1.56 μg/cm^3^); however, this hybrid was found to be much more toxic against Vero cell line (CC_50_ of 4.95 µM) [161]. In a similar way conjugates of gatifloxacin **92a**, **93** (R^1^ = OMe, R^2^ = Me), triazole, and isatin were obtained. Cycloadditions were performed in DMF giving the target compounds **92a** in 28–39% yield, while condensations with amine hydrochlorides gave the desired products **93** in 43–67% yield. All hybrids showed greater lipophilicity compared to gatifloxacin. The conjugates showed divergent antitubercular activity. Fluorine thiosemicarbazone (R = F, R^1^ = OMe; R^2^ = Me; R^3^ = NHCSNH_2_), chlorine methyloxime (R = Cl, R^1^ = OMe; R^2^ = Me; R^3^ = OMe), and fluorine methyloxime (R = F, R^1^ = OMe; R^2^ = Me; R^3^ = OMe) derivatives **93** were found to be much more active than the parent drug against both *M. tuberculosis* strains, H37Rv and multidrug resistant; although these compounds were much more toxic than free gatifloxacin [162, 163]. Moxifloxacin derivatives **92b** were synthesized in the same manner. The hybrids **94** were obtained by means of cycloaddition with Cu(OAc)_2_ in DMF followed by condensation reactions with amine hydrochlorides in 46–63% (**92b**) and 55–71% (**94**) yields, respectively. Most of the obtained compounds were less active against *M. tuberculosis* H37Rv and multidrug-resistant strains. The most active hybrid, fluorine methyloxime conjugate **94** (R = F, R^4^ = OMe), was, however, twice more potent than moxifloxacin. Nevertheless, this compound was extremely cytotoxic in Vero cell line. The metabolic stability and in vivo pharmacokinetic profiles of the most active conjugate was tested in mice after single oral administration of 50 mg/kg. The conjugate displayed much lower microsomal stability than the parent drug and inferior pharmacokinetic profile [164].

**Figure:**
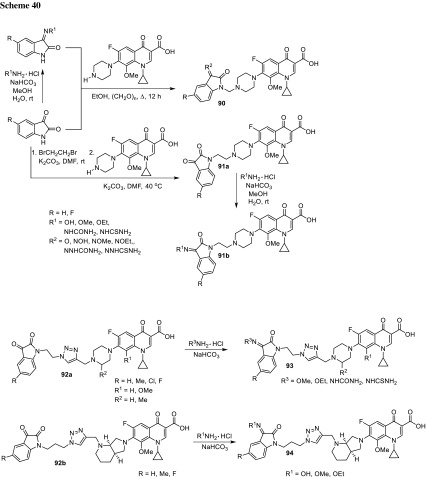

Figueroa-Valverde and coworkers synthesized dihydroxytestosterone–ciprofloxacin conjugate **95** in the reaction of ethylenediamine with fluoroquinolone to form an amide and subsequent coupling to dihydroxytestosterone hemisuccinate with EDCl to form the novel hybrid (Scheme 41). Since steroid derivatives may induce antibacterial effect [165] and cause membrane perturbation, the obtained conjugate was tested against *E. coli* and *S. aureus* bacterial strains. The novel hybrid **95** was found to be active against both pathogenic strains, but to the lesser extent than the parent drug ciprofloxacin [166].

**Figure:**
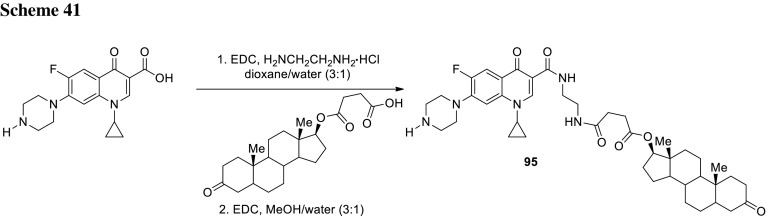

Fluoroquinolones possessing coumarin moiety **96** were synthesized by Guo and coworkers by nucleophilic substitution reaction of ciprofloxacin, 8-methoxyciprofloxacin or gatifloxacin with *α*-bromoketones or *α*-bromooximes (Scheme 42). The conjugates were tested in vitro for their antimycobacterial properties against *M. smegmatis* CMCC 93202 using serial double dilution technique in duplicate and *M. tuberculosis* H37Rv ATCC 27294 with the use of rapid direct susceptibility tests. The most active compounds were **96b** and **96n** [167].

**Figure:**
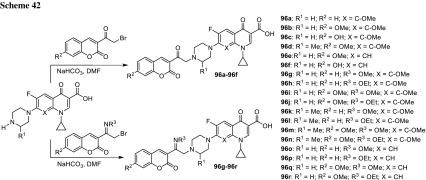

**Fig. 21:**
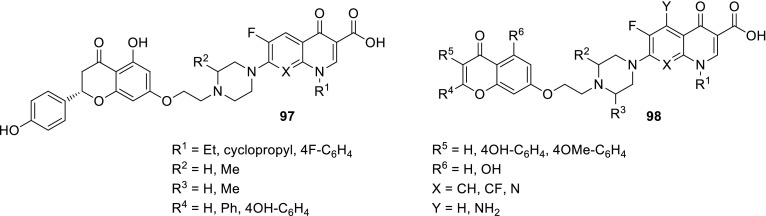
Structures of flavonoid–fluoroquinolone hybrids **97**–**98**

Flavonoids constitute a class of phenolic compounds widely found in herbs, seeds, fruits, and vegetables. Some of them, namely, naringenin [168], quercetin, kaempferol [169], chrysin [170], and genistein [171], were identified as effective inhibitors of multidrug transporters. Xiao and coworkers designed and obtained 21 fluoroquinolone–flavonoid hybrids as potential efflux pumps inhibitors by direct coupling of ciprofloxacin, sarafloxacin, norfloxacin, enrofloxacin, or lomefloxacin with naringenin, apigenin, genistin, chrysin, or formononetin with use of ethylene linker. The conjugates were tested against *S. aureus*, *B. subtilis*, *E. coli*, and *C. albicans.* Hybrids incorporating naringenin **97** were found to be the most active. Whole cells accumulation revealed that introduction of naringenin to the fluoroquinolone structure prevents of the hybrid from being the substrate for the efflux pumps. Ciprofloxacin (R^1^ = cyclopropyl, X = CH) and sarafloxacin (R^1^ = 4F-C_6_H_4_, X = CH) hybrids of naringenin (**97**) exhibited greater inhibitory activities than reference ciprofloxacin against the DNA gyrase in DNA supercoiling assay, confirming their strong fluoroquinolone character [172] (Fig. 21).

**Fig. 22:**
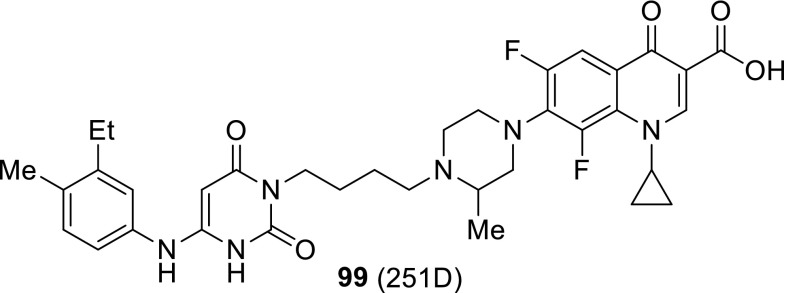
Structure of fluoroquinolone and anilinouracil hybrid **99** (251D)

DNA polymerase III C is an enzyme essential for bacterial DNA replication. It features an active site of a unique structure that can be selectively inhibited with anilinouracil-type dGTP analogs [173]. Recently a novel hybrid bearing fluoroquinolone and anilinouracil moieties, 251D (**99**), was prepared and evaluated biologically. 251D was found to be a highly selective potent inhibitor of DNA polymerase III C with little or no effect on Gram-positive and Gram-negative DNA polymerase III E as well as the mammalian polymerases α and γ. The inhibition effects for both topoisomerase and gyrase were lower than those of the parent fluoroquinolone and ciprofloxacin, but greater than nalidixic acid. This conjugate exhibited potent antibacterial and bactericidal activity against broad range of Gram-positive organisms. It also displayed rapid bactericidality within 2 h for *S. aureus* strains. Moreover, 251D exhibited more potency than an equimolar combination of the parent compounds, which indicates that fusion of anilinouracil and fluoroquinolone components into one molecule creates a synergistic effect, which is absent without the covalent linkage. The frequency of resistance development to this hybrid was lower than of the anilinouracil inhibitor and lower or similar to the patent fluoroquinolone after a single passage. Further tests revealed low toxicity on MRC-5 cell line (CC50 not less than 80 µg/cm^3^) [174] (Fig. 22).

**Fig. 23:**
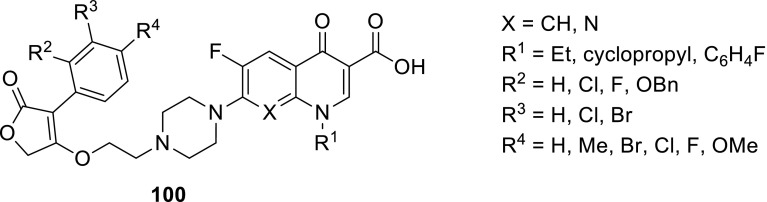
Structure of 3-arylfuran-2(5*H*)-one-fluoroquinolone conjugates **100**

3-Arylfuran-2(5*H*)-ones act as inhibitors of tyrosynyl tRNA synthetase (TyrRS), another bacterial enzyme [175] essential for bacterial protein synthesis. This enzyme is one of the aminoacyl-tRNA synthetases which ligates specific amino acids to their cognate tRNA molecules [176]. Wang and coworkers designed and synthesized 27 structures of 3-arylfuran-2(5*H*)-ones **100** merged with piperazinyl ring of fluoroquinolones. The hybrids were tested against *E. coli*, *B. subtilis*, and *S. aureus*, most of them were found to be more active than the reference drug, ciprofloxacin. The most potent compound **100** (R^1^ = cyclopropyl, R^2^ = R^3^ = H, R^4^ = F, X = CH) showed MIC values in the range of 0.09–0.19 µg/cm^3^. The inhibitory activities were measured against both possible target enzymes DNA gyrase and TyrRS to determine the possibility of a dual mode of action. The selected hybrids displayed similar or better effects against DNA gyrase than ciprofloxacin and significant inhibition effects against TyrRS. The best compound **100** (R^1^ = cyclopropyl, R^2^ = R^3^ = H, R^4^ = F, X = CH) was selected for docking studies at the active sites of TyrRS and DNA gyrase. In both cases it was proved to be tightly held in the binding pockets by several hydrogen-bonding interactions and hydrophobic contacts [177] (Fig. 23).

**Fig. 24:**
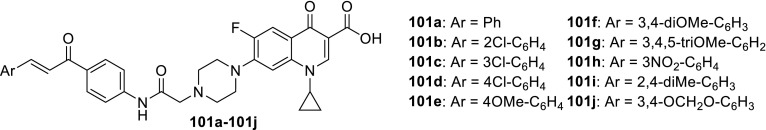
Structure of fluoroquinolone–chalcone hybrids **101**

Chalcones had been reported to inhibit cancer cell proliferation and induce apoptosis in various cell types [178]. Abdel-Aziz and coworkers prepared *N*-4-piperazinyl linked fluoroquinolone–chalcone hybrids **101** as potential cytotoxic agents in cancer therapy. They obtained novel conjugates by alkylation of ciprofloxacin with acylated chalcones in acetonitrile using trimethylamine as a base. The partition coefficient values for all the obtained hybrids were above the parent ciprofloxacin which may affect the cell permeability (log*P*_exp_ from − 0.0812 to 1.4684 vs. − 0.1432). Most of the obtained conjugates showed significant topoisomerase I and II inhibitory activity. Compounds **101a**, **101d**, **101e**, **101g**, **101j** were selected for in vitro anticancer screening performed by the National Cancer Institute against 60 cell lines from 9 tumor subpanels, including leukemia, melanoma, lung, colon, CNS, ovarian, renal, prostate, and breast cell lines. Hybrid **101a** exhibited the highest broad-spectrum antitumor activity, while **101g** revealed selectivity towards the leukemia subpanel  [179] (Fig. 24).

Vavrikova and coworkers prepared ciprofloxacin and norfloxacin conjugates of fluorine-containing hydrazones **102**. Substituted carbohydrazones are known for their good antitubercular activity [180]. Therefore, fluoroquinolone molecules bearing *N*-nucleophile moiety were combined with ethyl benzoylhydrazonoformates to afford the desired conjugated products (Scheme 43). The later were tested on mycobacterial strains. MIC values of ciprofloxacin conjugates were lower than both parent drugs. One of ciprofloxacin hybrids **102** bearing 4-fluorophenyl aryl substituent (R = cyclopropyl, Ar = 4F-C_6_H_4_) was subjected to stability test in aqueous buffers and rat plasma. The hybrid was stable at pH 7.4 and in an acidic buffer; however, in rat plasma slow decomposition was observed. Cytotoxicity of the obtained compounds was evaluated against human hepatocellular carcinoma cells (HepG2), peripheral blood mononuclear cells (PBMC), and human neuroblastoma cells SH-SY5Y. Selectivity index calculated for ciprofloxacin conjugates was extremely high, which make this type of hybrids promising potential antitubercular agents [181].

**Figure:**
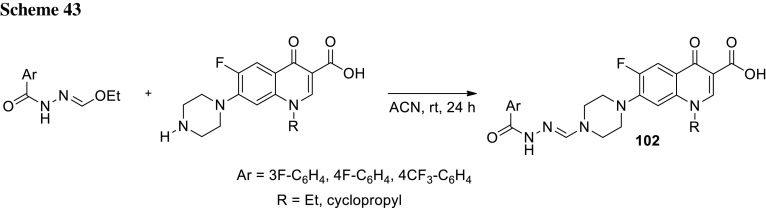

2-Aminobenzothiazole is one of the privileged structures in medicinal chemistry that demonstrate a myriad spectrum of biological activities such as anti-microbial, anti-inflammatory, analgesic and anti-tumor activity [182–184]. Sharma and coworkers reacted 2-(2-chloroacetylamino)-substituted benzothiazoles with ciprofloxacin, gatifloxacin, and norfloxacin, respectively, in DMF with use of sodium bicarbonate as a base (Scheme 44). The conjugates **103** were evaluated for their antibacterial activity by MIC method against *E. coli*, *P. aeruginosa*, *B. subtilis*, *Bacillus polymyxa*, and *S. aureus*. The most potent hybrid was ciprofloxacin conjugate **103** bearing the 6-chlorobenzothiazole substituent (R^1^ = cyclopropyl, R^2^ = R^3^ = R^4^ = R^6^ = H, R^5^ = Cl). This conjugate exhibited also significant analgesic activity, comparable to the standard drug diclofenac sodium, as tested in Swiss albino mice with the inhibition rates of 55.19 and 67.23% for the tested compound and the reference drug, respectively. Anthelmintic activity was evaluated against *Eisenia foetida*. The hybrids **103** showed promising anthelmintic properties at low concentrations as compared to reference drug, piperazine citrate, with mean paralysis time in the range of 22.80–32.60 vs. 34.4 min [185].

**Figure:**
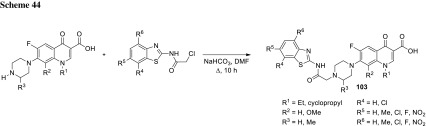

**Fig. 25:**
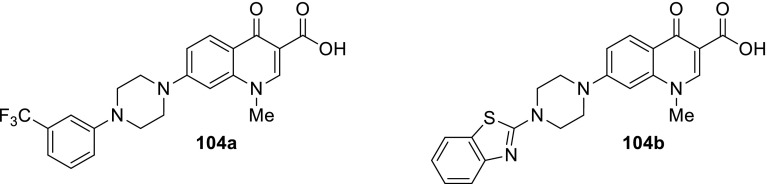
Structure of desfluoroquinolones **104a**, **104b**

6-Desfluoroquinolones (6-DFQs) interfere with the Tat-mediated transcription (TMT), a step of the HIV replicative cycle that is not targeted by any of the drugs currently in therapy. They are able to interact with the bulge of the HIV-1 TAR RNA element resulting in the Tat–TAR complex formation inhibition [186]. 6-DFQs derivatives **104** bearing benzothiazole or 2-trifluoromethylphenyl substituents were tested for their anti-human immunodeficiency virus (anti-HIV) activity. The novel compounds were found to inhibit HIV-1 replication and transcription in acutely and chronically HIV-1-infected as well as latently infected human primary monocytes/macrophages. The selectivity index for both compounds reached 125. Compound **104b** showed a pronounced suppressive effect on viral reactivation in vivo in SCID mice with no visible signs of drug toxicity [187] (Fig. 25).

**Fig. 26:**
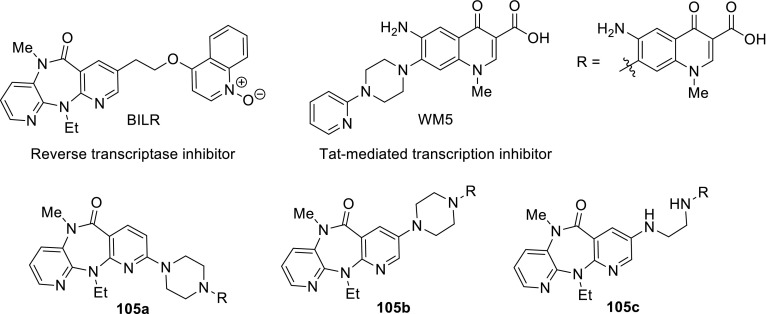
Structures of desfluoroquinolones **105a**–**105c**

Sancineto et al. have reported anti-HIV Designed Multiple Ligands (DMLs) merging the 6-DFQs to reverse transcriptase (RT) inhibitors, with the aim of blocking viral and cellular machineries in two viral cycle events taking place after and before viral integration, respectively. Compounds **105a**–**105c** were able to inhibit the RT, while hybrid **105a** showed activity against both targets, TMT and RT. The anti-HIV activity was tested against HIV-1 (IIIB) and HIV-2 (ROD) in acutely infected MT-4 cells, determining their cytotoxicity in parallel. All of the reported compounds were devoid of any antiviral activity. In particular, compound **105a**, despite showing inhibitory activity against both the targets, did not show anti-HIV activity in acutely infected cells at concentrations lower than those that were cytotoxic (CC_50_ = 23.5 μM). However, it proved that **105a** is able to selectively inhibit the HIV-1 reactivation from latently infected cells in in latently HIV-1 infected promyelocytic cells (OM-10.1) [188] (Fig. 26).

Pentafluoropyridine derivatives and cyanuric chloride were utilized for the synthesis of new piperazinyl-quinolone derivatives. The reactions were performed in DMF/water mixture in the presence of potassium carbonate giving rise to the formation of fluoropyridinyl and chloro-1,3,5-triazinyl piperazinylquinolone derivatives in good yields (Scheme 45). The synthesized compounds **106**–**108** were evaluated for their antibacterial activities against *Staphylococcus*, *Enterococcus*, *Escherichia*, *Proteus*, *Shigella*, and *Klebsiella* strains. The hybrids displayed improved antibacterial properties in comparison with ciprofloxacin. Compounds **106a** and **108a** showed good-to-excellent anti-microbial activity in agar disc diffusion method as well as with the use of broth microdilution technique [189].

**Figure:**
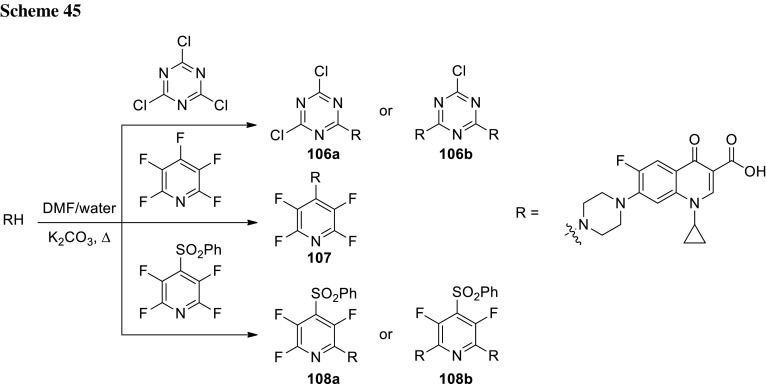

**Fig. 27:**
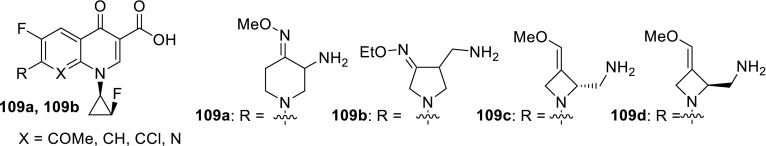
Structure of compounds **109a**–**109d**

Liu and coworkers introduced four-, five-, and six-membered nitrogen heterocyclic amine moieties to quinolone scaffold by displacement of C7 halogen atoms of fluoroquinolone derivatives bearing N1 2-fluorocyclopropyl substituent. They synthesized 27 novel fluoroquinolone conjugates. The obtained compounds were evaluated for their in vitro antibacterial activities on panel of Gram-positive and Gram-negative common pathogens. Most the synthesized hybrids exhibited good biological activity. Compounds **109a** (X = OMe) and **109b** (X = CH) were found to be potent antitubercular agents with MIC values for *M. tuberculosis* strains in the range of 0.0625–0.125 µg/cm^3^. In vivo activities of conjugates **109a** (X = OMe) and **109b** (X = N) was tested in mice model. The compound **109a** was found to be more potent than **109b**. However, due to poor solubility both compounds were less active than the parent fluoroquinolone [190] (Fig. 27).

## Conclusion

Quinolones represent an extremely interesting class of synthetic bactericides that can be exploited as precursors and building blocks for the synthesis of a wide range of organic molecules and coordination complexes, active pharmaceutical ingredients, and polymers. The huge number of publications which continuously describe novel methods to synthesize and to derivatize quinolones substrates account for their versatility and use in many fields of medicinal chemistry. Most of the methods for the synthesis of quinolone conjugates rely on the catalyzed and uncatalyzed coupling reactions. There are many promising directions in the application of novel quinolone conjugates in fields such as polymer engineering, biomaterials development and the design of novel hybrid bifunctional drugs. It is certain that quinolone modifications will continue to attract the attention of many research groups and that improvements in their biological potency as well as novel transformations of these compounds will be reported in the literature in the near future.
